# Computational gait analysis in the 3xTg-AD mouse model: evidence of progressive alterations in locomotor coordination

**DOI:** 10.3389/fnagi.2026.1879487

**Published:** 2026-09-23

**Authors:** Lidia Castillo Mariqueo, Martín Valdivia Gallardo, Lydia Giménez Llort, Carla Taramasco Toro, Lukas Chahuan Moyano, Diego Robles Cruz

**Affiliations:** 1 Faculty of Rehabilitation Sciences, School of Physical Therapy, Exercise and Rehabilitation Sciences Institute, Universidad Andres Bello, Santiago, Chile; 2 Facultad de Ingeniería, Universidad Andrés Bello, Instituto de Tecnología para la Innovación en Salud y Bienestar, Viña del Mar, Chile; 3 Institut de Neurociéncies, Universitat Autónoma de Barcelona, Barcelona, Spain; 4 Department of Psychiatry and Forensic Medicine, School of Medicine, Universitat Autónoma de Barcelona, Barcelona, Spain

**Keywords:** Alzheimer's disease, computational gait analysis, explainable AI, machine learning, murine model, pose estimation

## Abstract

**Introduction:**

Gait alterations have been proposed as functional indicators of neural dysfunction in Alzheimer's disease; however, their ability to capture disease-associated locomotor changes in preclinical models remains insufficiently characterized.

**Methods:**

In this study, we applied a neuroinformatics framework combining markerless pose estimation, kinematic feature extraction, conventional statistical analysis, supervised machine learning, and model interpretability to characterize gait alterations in the 3xTg-AD mouse model. Two complementary analyses were performed: cross-sectional genotype classification at 12 months of age in 46 mice (3xTg-AD, *n* = 22; 3xTg-WT, *n* = 24) and longitudinal assessment of 31 paired surviving animals re-evaluated at 16 months (3xTg-AD, *n* = 15; 3xTg-WT, *n* = 16).

**Results:**

At 12 months, gait features provided moderate-to-good genotype discrimination, with Logistic Regression achieving the highest balanced performance (AUC = 0.82) and showing relevant contributions from Duty Factor, Allometric Stride Length, Allometric Step Width, and Speed. In the longitudinal cohort, classification performance was more limited (Random Forest, AUC = 0.61), likely reflecting the combined influence of physiological aging, genotype-associated change, selective survival, and task re-exposure. Nevertheless, conventional statistical analysis and SHAP-based model interpretation consistently identified longitudinal changes in Allometric Stride Length and Normalized Coordination Instability as relevant contributors to genotype-associated locomotor divergence.

**Discussion:**

These findings suggest that computational gait analysis can capture relevant alterations in locomotor organization in the 3xTg-AD model, with cross-sectional differences associated with distributed spatiotemporal features and longitudinal changes showing a stronger contribution of stride- and coordination-related variables.

## 1 Introduction

Alzheimer's disease is a progressive neurodegenerative disorder classically characterized by cognitive decline and the accumulation of beta-amyloid plaques and neurofibrillary tau tangles (Scheltens et al., 2021). However, it is increasingly recognized as a disorder of distributed neural networks, in which non-cognitive manifestations may emerge early and contribute significantly to functional impairment (Seeley et al., 2009). Among these manifestations, subtle motor alterations, particularly changes in gait, have gained attention as potential indicators of neural dysfunction. Gait represents a complex functional phenotype that integrates motor control, balance, rhythm, postural adaptation, and higher-order cognitive processes. In humans, converging evidence indicates that the combined deterioration of gait and cognition is associated with an increased risk of dementia and with structural brain alterations, including hippocampal atrophy and white matter changes (Montero-Odasso et al., 2012; Karunarathna et al., 2024; Yan et al., 2025). Moreover, longitudinal studies have shown that changes in gait speed and variability may precede cognitive decline and predict the onset of dementia (Verghese et al., 2007; Beauchet et al., 2016). These findings have led to the conceptualization of gait not merely as a motor outcome, but as a cognitive–motor marker sensitive to disruptions in distributed neural networks.

From an applied perspective, gait analysis offers important advantages as a non-invasive and relatively low-cost functional assessment compared with many biological indicators. In this context, several studies have shown that gait analysis combined with machine learning can contribute to the discrimination of individuals with mild cognitive impairment, Alzheimer's disease, and healthy controls (Ghoraani et al., 2021; Varma et al., 2021). Complementarily, the use of wearable sensors and continuous monitoring has enabled the capture of locomotor patterns in ecological settings, potentially improving sensitivity for early detection and longitudinal assessment (Del Din et al., 2016; Weiss et al., 2016). Recent reviews further highlight that gait analysis using artificial intelligence represents a promising avenue for the development of non-invasive digital biomarkers, although important challenges remain regarding standardization, interpretability, cohort size, and model generalization (Al-Hammadi et al., 2024; Yan et al., 2025).

In animal models of Alzheimer's disease, accumulating evidence suggests that motor alterations may emerge during early or intermediate stages of the pathological process, sometimes before the appearance of overt cognitive deficits. Studies across different murine models have reported impairments in gait, coordination, and locomotor activity associated with neurodegenerative changes (Nyúl-Tóth et al., 2021; Wagner, 2019; O'Leary, 2018). These observations support the relevance of motor phenotyping as a complementary strategy for characterizing functional alterations in preclinical models. In this context, recent approaches have incorporated advanced computational techniques for gait analysis in animal models. Video-based methods and markerless motion estimation have enabled the capture of complex locomotor patterns and the quantification of alterations that may not be fully detected through traditional behavioral assessments (Bogachev, 2023). Furthermore, machine learning algorithms have shown potential for identifying discriminative kinematic features associated with neurodegenerative phenotypes, providing useful tools for classification and characterization of motor alterations (Huang, 2021).

In the preclinical domain, the 3xTg-AD mouse model represents a valuable platform for studying Alzheimer's disease-related alterations, as it recapitulates amyloid and tau pathology together with relevant behavioral and functional changes (Oddo et al., 2003; Giménez-Llort et al., 2007). In this model, functional limitations, spatiotemporal gait alterations, atypical locomotor patterns, and musculoskeletal impairments have been reported (Castillo-Mariqueo et al., 2021; Castillo-Mariqueo and Giménez-Llort, 2022b). However, many previous studies have relied primarily on global metrics or observational assessments, which may have limited sensitivity for detecting subtle alterations in motor control or for characterizing how multiple locomotor variables interact within a multidimensional phenotype.

Despite recent advances, a methodological gap remains between classical behavioral characterization and contemporary neuroinformatics approaches. In particular, few studies have integrated markerless pose estimation, multidimensional kinematic feature extraction, supervised classification, and explainable artificial intelligence within a unified framework to characterize gait alterations in Alzheimer's disease mouse models. Moreover, it remains unclear which biomechanical dimensions contribute to genotype discrimination and how these features change over time in longitudinally followed animals. In this context, the present study applies a neuroinformatics approach to analyze gait in 3xTg-AD mice, combining markerless computer vision, deep learning-based pose estimation, kinematic feature extraction, supervised machine learning, and model interpretability techniques. This framework was used to evaluate whether gait features contain sufficient information to discriminate between genotypes at 12 months of age and to explore longitudinal gait changes in a paired surviving cohort re-evaluated at 16 months. From this perspective, gait is considered as a computational phenotype that may provide complementary information for characterizing locomotor alterations in preclinical models of Alzheimer's disease.

## 2 Materials and methods

### 2.1 Experimental design and definition of experiments

The study was structured into two complementary experiments aimed at characterizing gait as a computational motor phenotype in the 3xTg-AD mousse model. The first experiment evaluated the ability of gait features to discriminate between genotypes at 12 months of age, whereas the second explored longitudinal gait changes in the paired surviving cohort re-evaluated at 16 months.

Triple transgenic Alzheimer's disease mice [3xTg-AD; PS1(M146V), APP(Swe), and tau(P301L)] and their non-transgenic controls (3xTg-WT), of both sexes, were evaluated at 12 and 16 months of age. These ages correspond to intermediate and advanced stages of the experimental model. Locomotor activity, exploratory behavior, and cognitive status have been previously characterized in this model using standardized behavioral tests (Castillo-Mariqueo et al., 2021; Castillo-Mariqueo and Gimenez-Llort, 2022). In addition, previous work has reported early behavioral alterations resembling BPSD-like symptoms, as well as cognitive deficits involving learning, short- and long-term memory, and behavioral flexibility. During gait data acquisition, no severe motor deficits or sensory impairments were observed that could interfere with task performance.

#### 2.1.1 Experiment 1: Genotype classification at 12 months

Supervised machine learning models were trained to discriminate between non-transgenic control mice (3xTg-WT) and AD model mice (3xTg-AD) at 12 months of age, including both males and females. This analysis aimed to evaluate whether gait features contained sufficient information to distinguish between genotypes at an intermediate stage of the model.

#### 2.1.2 Experiment 2: Longitudinal gait changes following task re-exposure

Changes in gait patterns were evaluated at 16 months in animals that had been previously assessed at 12 months and were re-evaluated under the same experimental conditions after re-exposure to the locomotor task. This analysis was designed to characterize longitudinal kinematic changes in the surviving paired cohort (*N* = 31; 3xTg-AD, *n* = 15; 3xTg-WT, *n* = 16) and to explore whether these changes differed between 3xTg-AD and 3xTg-WT mice. Because the design did not include an independent non-re-exposed control group, Experiment 2 was interpreted as an exploratory longitudinal analysis of the paired surviving cohort.

### 2.2 Age equivalence and body weight

To contextualize the translational relevance of the selected time points, an approximate equivalence between murine and human age was considered. In general terms, mice aged between 12 and 16 months correspond to an approximate range of 50 to 70 years in humans (Dutta and Sengupta, 2016; Flurkey et al., 2007). This interval spans from middle age to advanced stages of aging and is relevant for the study of Alzheimer's disease-related functional alterations. In this work, two time points within this range were selected: 12 months, representing an intermediate stage of the model, and 16 months, corresponding to a more advanced age. This selection allowed the evaluation of genotype-related gait differences at 12 months and the exploratory assessment of longitudinal gait changes in animals re-evaluated at 16 months.

Figure 1 illustrates the approximate correspondence between murine and human age, providing a reference framework for interpreting the selected time points in a translational context. This approximation acknowledges the inherent limitations of cross-species comparisons. Additionally, body weight was recorded prior to each experimental session to characterize the cohort and account for potential effects of body mass on locomotor parameters. These measurements were used to contextualize gait differences and to support the allometric normalization of spatial gait variables (Table 1).

**Figure 1:**
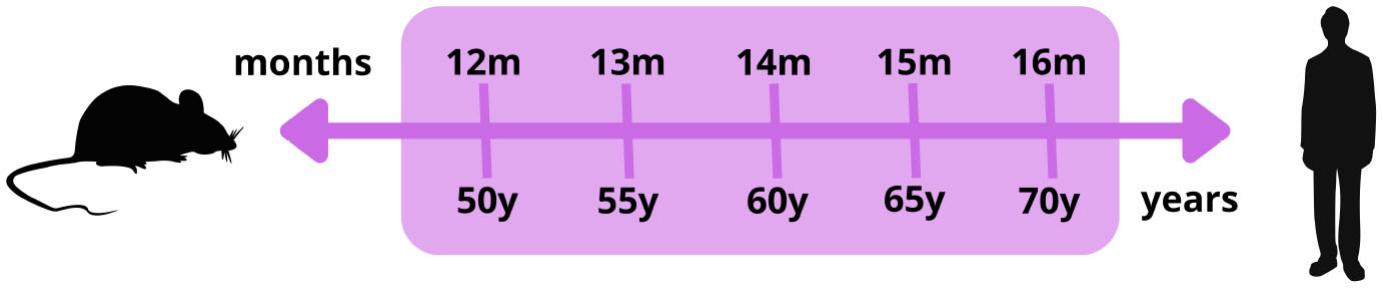
Approximate equivalence between murine age (12–16 months) and human age (50–70 years), providing a reference framework for the translational context of the experimental design.

**Table 1:** Body weight by sex and genotype at 12 months of age (M12), 16 months of age (M16), and longitudinal change (Δ).

Group	*n* M12	M12 (mean ±SEM)	*n* M16	M16 (mean ±SEM)	*n* paired	Δ (mean ±SEM)	RIP at M16
Male 3xTg-AD	19	35.36 ± 1.84	16	34.07 ± 1.62	16	-1.24 ± 1.32	3
Male 3xTg-WT	12	29.48 ± 0.58	8	28.96 ± 0.45	8	-0.82 ± 0.40	4
Female 3xTg-AD	14	33.93 ± 2.14	9	26.62 ± 0.75	9	-6.62 ± 2.69	3
Female 3xTg-WT	18	27.08 ± 1.06	12	25.65 ± 0.78	12	-1.83 ± 1.15	6

Values are reported as mean ± standard error of the mean (SEM). RIP indicates animals lost before the 16-month assessment.

### 2.3 Gait video acquisition

Gait recordings were acquired during a one-minute period of free locomotion within a transparent acrylic enclosure (27.5 × 9.5 cm). A single fixed camera, positioned directly below the transparent walkway, captured a ventral view of the animal at a resolution of 1920 × 1080 pixels and a constant frame rate of 30 frames per second (fps). This bottom-view configuration enabled simultaneous visualization of the four limb contact points and was used to extract two-dimensional gait kinematics from the ventral plane. Although this setup reduces lateral occlusion and facilitates the tracking of paw contact points, it does not capture out-of-plane movements or three-dimensional limb trajectories. To ensure consistent temporal analysis and maximize the visual contrast between the paws and the walkway background, recordings were performed under controlled white LED illumination directed upward through the transparent surface.

### 2.4 Pose estimation and gait digitization

Gait digitization was performed using markerless pose estimation with the DeepLabCut framework, a deep learning-based tool widely used and validated for quantitative analysis of animal behavior (Mathis et al., 2018; Nath et al., 2019). DeepLabCut employs deep convolutional neural networks to identify and track user-defined anatomical keypoints directly from video sequences, without requiring physical markers or specialized equipment. In the present study, this framework was applied to ventral-view recordings to extract two-dimensional anatomical keypoint trajectories for subsequent gait kinematic analysis.

#### 2.4.1 Definition of anatomical keypoints for gait characterization

A set of anatomical keypoints relevant for gait characterization was defined, including the snout, tail base, left and right forelimbs, and left and right hindlimbs. The selection of these keypoints allowed the capture of both global body kinematics and limb-specific dynamics during locomotion. To minimize labeling variability, standardized anatomical references were used. The snout was defined based on the nasal tip, while limb positions were defined using the paw pads, corresponding to the effective contact points with the surface. To support anatomical consistency and positional accuracy in the training dataset, all keypoints were manually annotated by a domain expert with biological expertise. The six defined keypoints and their anatomical placement are illustrated in Figure 2.

**Figure 2:**
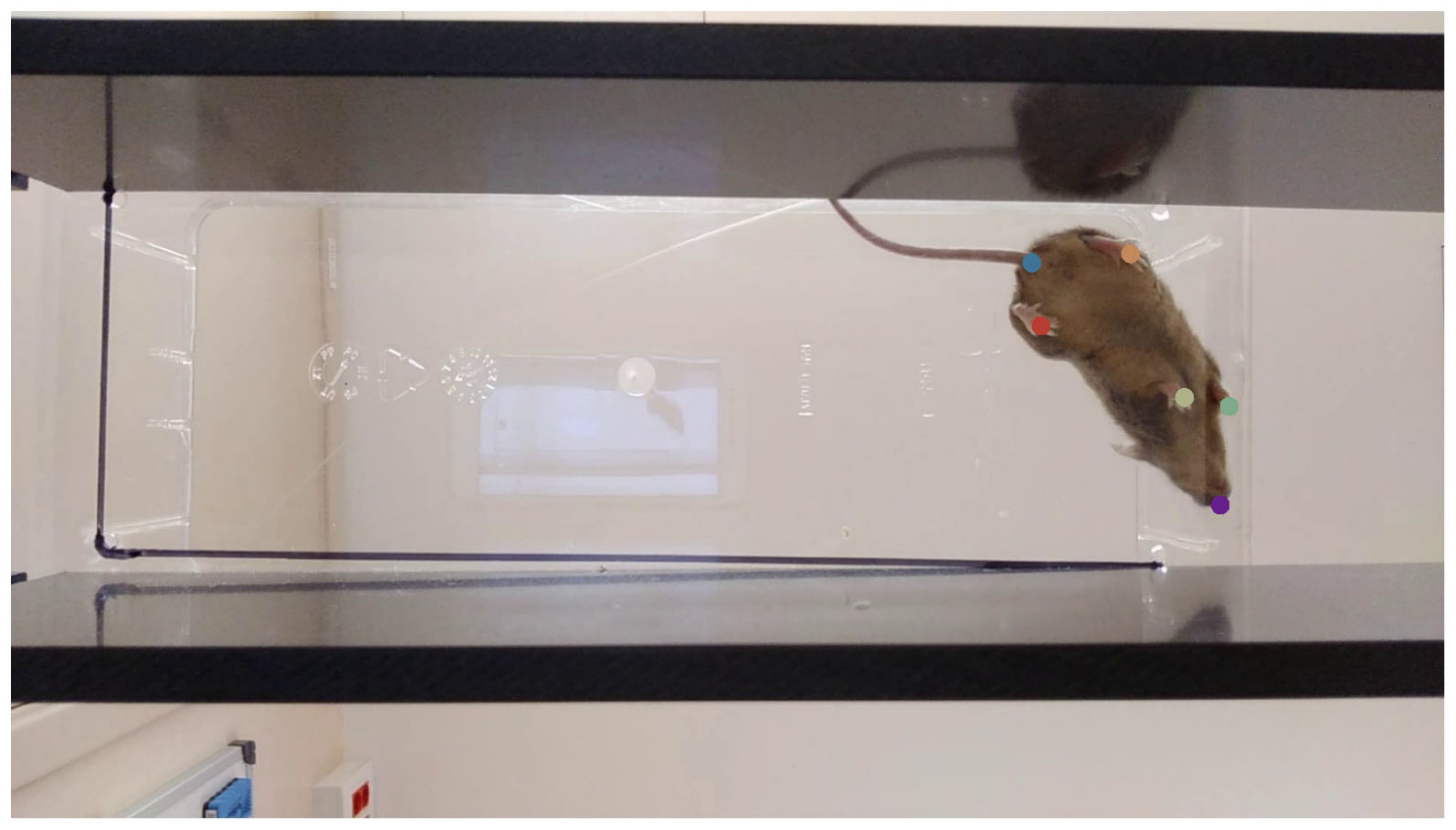
Representative ventral-view frame illustrating the six anatomical keypoints defined for markerless pose estimation and their associated marker colors: Snout (nasal tip, purple), Tail Base (tail-base region, blue), left forelimb (FL-L, pale green), right forelimb (FL-R, mint green), left hindlimb (HL-L, red), and right hindlimb (HL-R, orange). Limb keypoint positions correspond to paw pad contact points for all four limbs. Colored markers indicate the keypoint locations shown for visualization.

#### 2.4.2 Frame selection and labeling

For training the pose estimation model, a subset of frames was extracted from six independent gait recording sessions, balanced by genotype (three 3xTg-AD and three 3xTg-WT control sessions). To increase the visual diversity of captured postures and improve coverage across different phases of the gait cycle, frame selection was performed using a *k*-means clustering algorithm implemented in DeepLabCut (Mathis et al., 2018; Nath et al., 2019). A total of 50 frames were extracted per video, yielding a consolidated training dataset of 300 frames.

Manual annotation was subsequently performed by a single domain expert to ensure anatomical consistency, assigning two-dimensional coordinates (*x, y*) to each of the six predefined anatomical keypoints. To avoid introducing *post-hoc* observer bias into the full tracking dataset, no manual correction was applied to the model's inference outputs after automated pose estimation. Instead, tracking quality was managed using the predictive confidence threshold and the physiological velocity filter detailed in subsection 2.5.1. Finally, the labeled dataset was partitioned into a training subset (95%) and a hold-out test subset (5%) to evaluate out-of-sample localization error.

### 2.5 Data preprocessing and processing pipeline

Raw trajectories obtained from the DeepLabCut network were processed through an automated sequential pipeline. This workflow was designed to reduce positional tracking noise, manage missing coordinate values, and standardize the extracted biomechanical metrics according to the methodological steps described below.

#### 2.5.1 Coordinate extraction, noise thresholding, and artifact filtering

Pose estimation and coordinate extraction (*x, y*) were performed using the DeepLabCut neural architecture (Mathis et al., 2018; Nath et al., 2019). To extract reliable kinematic time series and reduce tracking noise without discarding informative limb trajectories, a dual-thresholding procedure was implemented.

First, a predictive confidence threshold (*p-cutoff* ) was established. Regarding predictive confidence, the likelihood scores output by the network exhibited an asymmetric distribution across the full dataset (*n* = 116, 412 predictions). As illustrated in Figure 3, 45.9% of frames yielded high-confidence scores (*p*≥0.8), while 25.4% fell below *p* = 0.3. This low-confidence tail corresponded predominantly to frames affected by motion blur and partial occlusion during fast limb swing phases. Per-keypoint analysis revealed substantial heterogeneity in tracking reliability. Axial landmarks, such as the Tail Base ($\bar{p}=0.742\pm 0.366$) and Snout ($\bar{p}=0.663\pm 0.404$), achieved higher confidence than distal limb keypoints. In contrast, the forelimb keypoints, particularly the left forelimb ($\bar{p}=0.367\pm 0.218$), exhibited the lowest mean likelihood, consistent with the greater occlusion and motion blur associated with rapidly moving distal landmarks in ventral-view recordings.

**Figure 3:**
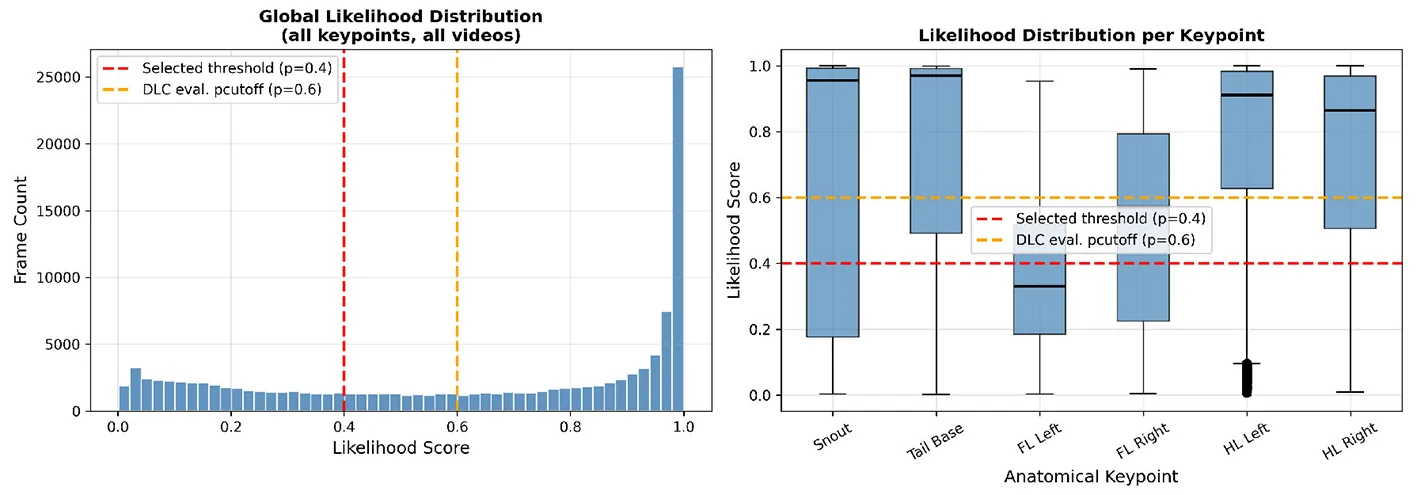
Distribution of DeepLabCut predictive confidence scores (*p*). The **left panel** shows the global distribution of confidence scores across all frames, while the **right panel** illustrates variability in predictive confidence across anatomical keypoints, highlighting the lower confidence associated with distal forelimb landmarks.

To address this heterogeneity, the selection of the confidence parameter was empirically justified through a systematic data quality analysis comparing four threshold conditions: *p* = 0.4 (with physiological velocity filtering), *p* = 0.5, *p* = 0.6 (the threshold at which the model achieved its reported test RMSE), and *p* = 0.7. Pre-interpolation diagnostics revealed a progressive loss of tracking data as the confidence threshold increased (Figure 4). Specifically, applying *p*≥0.6 resulted in 45,516 unrecoverable frames after linear interpolation and caused 24 out of 46 animals (52.2%) to lose the Coordination Instability measurement. At *p* = 0.7, this loss increased to 40 animals (87.0% of the cohort). In contrast, the threshold of *p*_cutoff_ = 0.4 combined with physiological velocity filtering resulted in 23,694 unrecoverable frames (10.2%) and preserved complete Coordination Instability data for 43 of the 46 animals. A sensitivity analysis showed that the direction of group effects for Coordination Instability remained consistent across all four evaluated thresholds, supporting the use of *p* = 0.4 to balance noise reduction with the preservation of forelimb kinematics. A complete numerical summary of all quality metrics across the four evaluated thresholds is provided in Table 2.

**Figure 4:**
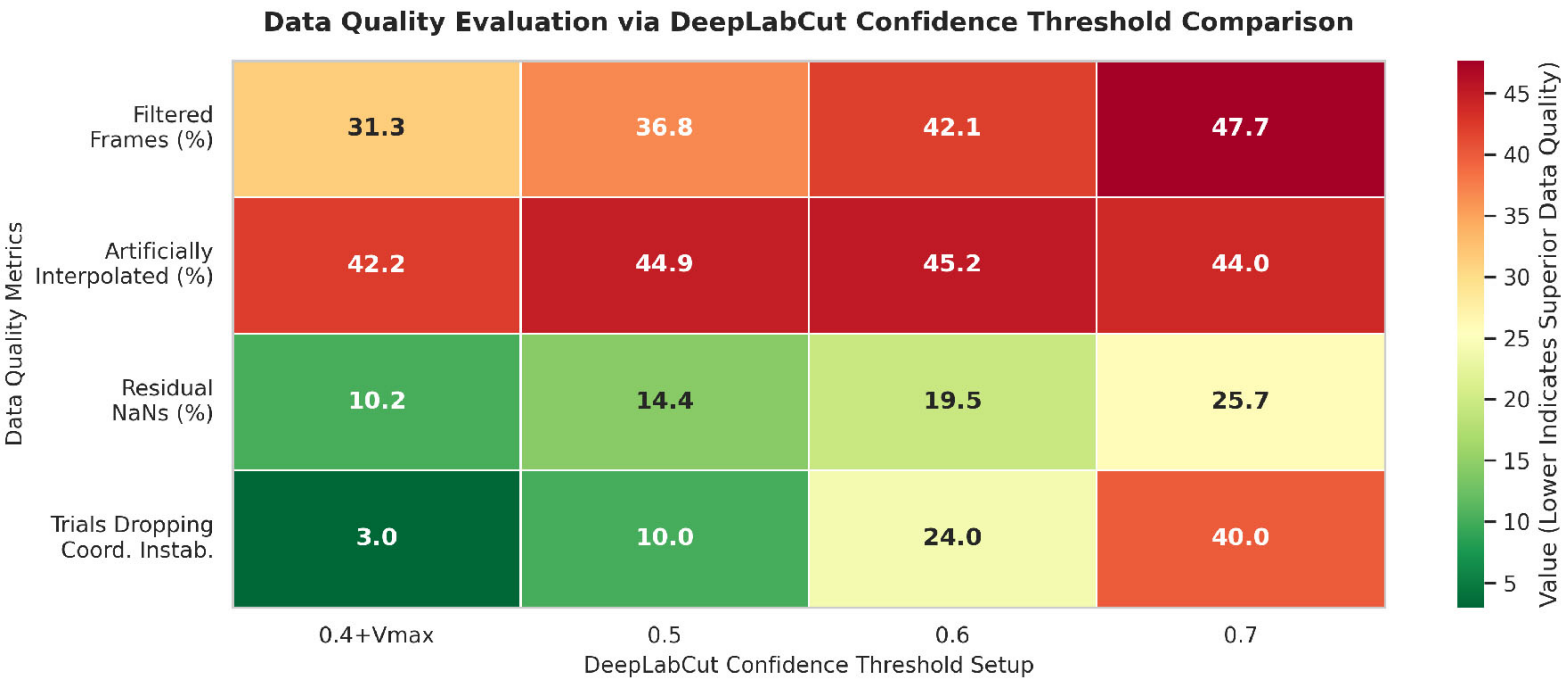
Data quality comparison across DLC confidence thresholds (0.4 + V_*max*_, 0.5, 0.6, and 0.7). Metrics shown are: percentage of frames filtered by the confidence criterion before interpolation, percentage of interpolated frames, percentage of residual NaN frames after interpolation, and number of animals losing Coordination Instability data. Color scale ranges from green (better quality) to red (lower quality). The threshold of 0.4 + V_*max*_ showed the lowest residual NaN rate (10.2%) and preserved complete Coordination Instability data for 43 of the 46 animals, supporting its use as the primary analysis threshold.

**Table 2:** Data quality comparison across DLC confidence thresholds.

Threshold	Filtered frames (%)	Interpolated frames (N)	Residual NaNs (%)	Animals losing CI	*V*_*max*_ artifacts
0.4 + *V*_*max*_ (selected)	31.3%	49,126	**10.2%**	**3 / 46**	306
0.5	36.8%	52,322	14.4%	10 / 46	—
0.6 (DLC eval.)	42.1%	52,572	19.5%	24 / 46	—
0.7	47.7%	51,168	25.7%	40 / 46	—

CI, coordination instability; *V*_*max*_, physiological velocity filter; total dataset: 19,402 frames from 46 animals.

Second, to complement the selected confidence threshold and exclude residual tracking artifacts that passed the likelihood criterion but represented biologically implausible displacements, a physiological velocity filter was applied. Frame-to-frame displacements implying instantaneous velocities exceeding a predefined maximum threshold (*V*_*max*_) were discarded from the velocity time series prior to kinematic computation. *V*_*max*_ was formally defined as:


$$\begin{array}{l} V_{max}=3000\text{px/s} \end{array}$$ (1)

Under the calibration parameters of the recording system (κ = 46.20 px/cm), this value corresponds to approximately 65 cm/s, representing a conservative upper bound consistent with reported maximum sprint speeds in laboratory mice (Castillo-Mariqueo et al., 2021). Displacements exceeding this limit were classified as tracking artifacts and excluded. This fixed-boundary approach removed 306 artifact frames across the dataset and was selected over conventional spatial outlier filtering based on the global Interquartile Range (IQR). A global IQR-based criterion was considered potentially problematic, as it could classify extreme but potentially meaningful displacements (such as stumbles, limps, or episodic dragging) as noise, thereby attenuating biologically relevant genotype-associated locomotor variation (Castillo-Mariqueo and Giménez-Llort, 2022a).

Where IQR-based outlier removal was retained for feature-level quality control, it was applied conservatively and only after feature aggregation. Finally, kinematic aggregates per recording session were computed using the median rather than the arithmetic mean, providing summary statistics that were less sensitive to residual spatial anomalies.

#### 2.5.2 Trajectory reconstruction and missing data imputation

To reduce tracking noise associated with the selected confidence threshold and to recover short gaps in coordinate trajectories, linear interpolation was applied to the temporal series of each anatomical keypoint. To avoid generating artificial trajectories across prolonged tracking failures, interpolation was strictly limited to a maximum of 5 consecutive frames, equivalent to approximately 166 ms of recording at 30 fps. Any occlusion or tracking loss exceeding this temporal limit was retained as a null value to preserve the structure of the original kinematic signal.

Subsequently, during the predictive modeling phase on the consolidated feature matrices, any remaining null values were imputed within the cross-validation pipeline. For each fold, imputation parameters were estimated exclusively from the training data and then applied to the corresponding held-out test data. This procedure ensured that information from the test fold was not used during preprocessing and reduced the risk of data leakage during model evaluation.

### 2.6 Extraction of gait kinematic features

From the processed spatiotemporal trajectories of anatomical keypoints, a set of kinematic features was derived to characterize gait as a multidimensional phenomenon. These metrics integrated spatial, temporal, and coordination-related components and were selected based on their documented biomechanical relevance in Alzheimer's disease murine models (Castillo-Mariqueo et al., 2021; Castillo-Mariqueo and Giménez-Llort, 2022b). All variables were computed from coordinate time series expressed in real-world metric units following pixel-to-metric conversion and were aggregated to a single representative value per animal per session using the median, reducing sensitivity to residual tracking artifacts. Spatial parameters were additionally normalized by body mass using allometric scaling to account for body-mass-related morphological differences between groups. The feature space encompassed global locomotor parameters, spatial metrics of gait pattern, a temporal descriptor of the locomotor cycle, and a coordination-related instability metric. The operational definition, computational basis, and units of each feature are provided in Table 3. This nine-dimensional feature matrix constituted the input for all supervised machine learning classifiers.

**Table 3:** Operational definitions of the nine kinematic and allometric features constituting the input feature matrix for machine learning classification.

**Metric**	Equation	Operational definition
Pixel-to-metric conversion	$\kappa =\frac{L_{r}}{L_{p}}$	Scaling factor derived from a physical reference object of known length (*L*_*r*_) measured in pixels (*L*_*p*_) within the video frame, used to convert all coordinate data from pixels to centimeters.
Gait Speed	*v* = median_*t*_(*v*_*t*_)	Median of frame-to-frame Euclidean displacements of the tail base keypoint, expressed in cm/s after pixel-to-metric conversion, where *v*_*t*_ = κ·||**x**_*t*_−**x**_*t*−1_||·*f*_*ps*_. Frames exceeding the physiological velocity limit *V*_*max*_ (Equation 1) are excluded prior to aggregation.
Step Width	*W* = median_*t*_(κ·|*y*_*L, t*_−*y*_*R, t*_|)	Median lateral distance along the transverse axis between the left and right hindlimb keypoints across all valid frames of the recording session, representing the base of support.
Stride Length	*S* = median_*t*_(κ·||**x**_*t*_−**x**_*t*−1_||)	Median frame-to-frame displacement of the tail base keypoint across all valid frames, expressed in cm. This metric provides an approximation of locomotor displacement magnitude under continuous free-movement conditions.
Duty Factor	$DF=\frac{N_{stance}}{N_{total}}$	Proportion of total valid frames in which the instantaneous velocity of the reference hindlimb (*v*_*t*_) falls below a stance-phase velocity threshold (*v*_*stance*_ = 2.0 cm/s), approximating the fraction of the locomotor cycle spent in ground contact.
Tortuosity Index	$TI=\frac{\overset{N-1}{\underset{i=1}{\sum }}||x_{i+1}-x_{i}||}{\left||x_{N}-x_{1}|\right|}$	Ratio of the total path length traversed by the tail base keypoint to the net straight-line displacement between the first and last valid positions. Values approaching 1.0 indicate straighter trajectories, whereas higher values indicate greater path irregularity.
Coordination Instability (SD)	$CI=\mathrm{std}\left(r_{t}^{\left(\Delta t\right)}\right),r_{t}^{\left(\Delta t\right)}=\mathrm{corr}\left(v_{FL,t:t+\Delta t},v_{HR,t:t+\Delta t}\right)$	Standard deviation of the rolling Pearson correlation (Δ*t* = 15 frames, ≈500 ms at 30 fps) between the instantaneous velocity magnitudes of the diagonal limb pair (left forelimb – right hindlimb). Higher values indicate greater temporal variability in interlimb coordination.
Normalized Coordination Instability (Ratio)	$\hat{CI}=\frac{CI}{\bar{r}}$	Coordination Instability normalized by the mean absolute rolling correlation $\bar{r}$, providing a dimensionless ratio that accounts for differences in baseline coupling strength between animals.
Allometric Step Width	$Ŵ=\frac{W}{M^{1/3}}$	Step width normalized by the cube root of body mass *M* (g), following allometric scaling for linear skeletal dimensions. This normalization reduces the influence of body size on lateral gait parameters.
Allometric Stride Length	$Ŝ=\frac{S}{M^{1/3}}$	Stride length normalized by the cube root of body mass *M* (g), following the same allometric convention as Step Width.

All spatial metrics are expressed in centimeters following pixel-to-metric conversion (κ).

Aggregation across frames was performed using the median to reduce sensitivity to residual tracking artifacts.

*f*_*ps*_, frames per second (30 fps); *V*_*max*_: physiological velocity limit (Equation 1); *M*: body mass in grams; *v*_*FL*_: left forelimb velocity; *v*_*HR*_: right hindlimb velocity; Δ*t*: rolling window of 15 frames.

### 2.7 Conventional statistical analysis

Prior to machine learning classification, conventional statistical analyses were performed to characterize group-level differences in gait features across both experiments. Normality of the data distributions was assessed using the Shapiro-Wilk test. Given the limited sample sizes and the non-normal distribution of several variables, non-parametric tests were applied to all comparisons.

Effect sizes were quantified using two complementary estimators: Cohen's *d* (standardized mean difference) and the rank-biserial correlation (*r*_*rb*_), the latter providing a non-parametric measure of effect magnitude. To provide uncertainty estimates under small sample conditions, 95% confidence intervals (CI) for group medians were computed via bootstrap resampling (2,000 iterations). All statistical tests were two-sided, with the significance threshold set at α = 0.05.

### 2.8 Experiment 1 (cross-sectional, *N* = 46)

Group-level differences in the nine extracted gait features were assessed between 3xTg-AD (*n* = 22) and 3xTg-WT control (*n* = 24) mice at 12 months of age using Mann-Whitney U tests. Each feature was tested independently to characterize univariate group-level differences prior to multivariate classification.

### 2.9 Experiment 2 (longitudinal, *N* = 31 paired animals)

Two complementary analyses were conducted on paired longitudinal data, with measurements available at both 12 and 16 months. First, within-group temporal changes were assessed using the Wilcoxon Signed-Rank test, a non-parametric paired test appropriate for repeated measures on the same subject. Effect sizes for within-group changes were quantified using the rank-biserial *r* statistic ($r=Z/\sqrt{N}$). Second, to assess whether the magnitude of change differed between genotypes, Mann-Whitney U tests were applied to the between-timepoint delta values (Δ = value_16*m*_−value_12*m*_), comparing 3xTg-AD and 3xTg-WT control groups. Effect sizes for between-group delta comparisons were quantified using Cohen's *d* and the rank-biserial correlation. This two-level analysis was used to characterize within-group temporal changes and between-genotype differences in longitudinal change, providing conventional statistical context for the paired cohort.

This analytical pipeline was applied independently to each experiment and was designed to complement the multivariate machine learning classification framework by providing conventional statistical context for the model's feature contribution rankings. All computations were performed using the SciPy ecosystem in Python (Virtanen et al., 2020).

### 2.10 Supervised classification models and validation

A diverse set of machine learning algorithms was evaluated to classify gait patterns, encompassing ensemble and gradient boosting methods (XGBoost, Extra Trees, LightGBM, CatBoost, Random Forest, and AdaBoost), probabilistic and distance-based approaches (Logistic Regression, Naive Bayes, SVM, and KNN), and neural architectures (Multilayer Perceptron, MLP). This selection allowed comparison of predictive performance across different inductive biases and decision structures, facilitating the identification of the model selected for subsequent interpretation.

To reduce the risk of overfitting inherent to small murine cohorts, model training and evaluation relied on a stratified cross-validation strategy rather than a static data split. The feature matrices were previously structured to yield exactly one independent observation per animal—specifically, one median-aggregated feature vector per recording session in Experiment 1, and one longitudinal delta (Δ) vector per paired subject in Experiment 2—which precluded the necessity for group-constrained folding. Therefore, a standard 5-fold stratified cross-validation scheme (*k* = 5, shuffle=True, random_state=42) was implemented. This approach preserved the class distribution (3xTg-AD and 3xTg-WT) across all training and test splits. Classification probabilities were estimated only when a sample belonged to the held-out test fold, generating a complete set of out-of-fold (OOF) predictions. Performance metrics and ROC curves were then computed from these OOF predictions to maintain rigorous separation between training and test evaluations (Hastie et al., 2009).

To further reduce the risk of data leakage, feature preprocessing steps, including standard scaling and missing-value imputation, were encapsulated within the cross-validation pipelines. Under this framework, imputation and scaling parameters were estimated exclusively from the training folds and subsequently applied to the corresponding held-out test data. Hyperparameter optimization was performed using grid search nested within the pipeline architecture, with Macro F1 score used as the optimization objective to account for class imbalance. The area under the receiver operating characteristic curve (AUC) was computed as the primary global evaluation metric due to its threshold-independent nature (Fawcett, 2006).

The complete hyperparameter search space explored during optimization is detailed in Supplementary Tables S1, S2. The selected hyperparameter configurations for each model are reported in Supplementary Table S3 for Experiment 1 and Supplementary Table S4 for Experiment 2.

### 2.11 Model interpretability

To interpret the contribution of individual gait features to model predictions, explainable artificial intelligence (XAI) techniques based on SHapley Additive exPlanations (SHAP) were implemented (Lundberg and Lee, 2017). SHAP values were used to quantify how each kinematic feature contributed to the output of the selected predictive models within each experimental setting.

For Experiment 1, the selected Logistic Regression model afforded direct interpretability through its learned coefficient vector. Each coefficient quantified the signed contribution of the corresponding kinematic feature to the log-odds of the 3xTg-AD class. SHAP values for the Logistic Regression model were computed from these coefficients using the out-of-fold (OOF) predictions generated during stratified cross-validation, so that interpretability was based on held-out model behavior rather than on the training data alone (Lundberg et al., 2020).

For Experiment 2, the selected Random Forest model corresponded to a tree-based ensemble architecture, for which the TreeSHAP algorithm was employed (Lundberg et al., 2020). TreeSHAP is optimized to compute Shapley values for tree-based models and was applied to the OOF predictions generated during cross-validation, preserving consistency with the validation framework used throughout the classification pipeline.

In both experiments, SHAP values were summarized from two complementary perspectives. At the global level, the mean absolute magnitude of SHAP values was calculated to rank features according to their contribution to model output. At the local level, directional analysis was used to evaluate how higher or lower values of a given metric shifted the predicted probability toward the 3xTg-AD or 3xTg-WT class. Importantly, SHAP values quantify feature contributions within the predictive model and should not be interpreted as evidence of direct biological causality. Their interpretation is therefore restricted to the model's decision structure and to the dataset analyzed in this study (Lundberg and Lee, 2017).

### 2.12 Computational environment and software dependencies

Computational analyses were conducted using a hybrid environment that combined cloud-based and local computing resources. Deep learning-based markerless pose estimation and anatomical keypoint extraction were performed on a Google Colaboratory (Colab) instance equipped with an NVIDIA A100 Tensor Core GPU, using DeepLabCut (v. 3.0.0rc13) and the tensorpack library (v. 0.11).

Subsequent machine learning classification, statistical evaluation, and model interpretability analyses were performed on a CPU-based cloud instance. This phase of the analytical pipeline was executed in Python (v. 3.12.13). Data wrangling and numerical operations were conducted using pandas (v. 2.2.2) and NumPy (v. 2.0.2). Supervised learning algorithms were implemented using scikit-learn (v. 1.6.1) (Pedregosa et al., 2011), XGBoost (v. 3.2.0) (Chen and Guestrin, 2016), CatBoost (v. 1.2.10) (Prokhorenkova et al., 2018), and LightGBM (v. 4.6.0) (Ke et al., 2017). Model interpretability was computed using the shap library (v. 0.51.0).

Data consolidation, code development, and the generation of high-resolution kinematic and statistical visualizations were managed locally on a workstation equipped with an Intel Core i5-1135G7 processor (2.40 GHz) and 8 GB of RAM, running a 64-bit Windows 11 Pro operating system. Visualizations were generated using *matplotlib* (v. 3.10.0) and *seaborn* (v. 0.13.2).

## 3 Results

### 3.1 Univariate analysis of cross-sectional features (12 months)

Univariate analysis of the nine cross-sectional gait features at 12 months (Figure 5) revealed significant group-level differences mainly in spatial base-of-support and temporal phase-distribution metrics. Specifically, 3xTg-AD mice exhibited a wider Step Width (*p* = 0.0031, |*d*| = 0.967) and an increased Duty Factor (*p* = 0.0018, |*d*| = 0.835) compared with wild-type controls. After allometric normalization by body mass, significant differences were also observed for Allometric Step Width (*p* = 0.034, |*d*| = 0.535) and Allometric Stride Length (*p* = 0.034, |*d*| = 0.701), suggesting genotype-associated spatial differences after accounting for inter-individual variation in body mass. In contrast, global locomotor velocity, path irregularity, and interlimb Coordination Instability did not differ significantly between groups (*p*>0.05) at this age. Overall, Step Width and Duty Factor showed the largest univariate effect sizes and were consistent with the most informative predictors identified in the subsequent multivariate interpretation. However, no single cross-sectional metric appeared sufficient to separate the groups in isolation, supporting the use of a multivariate machine learning framework.

**Figure 5:**
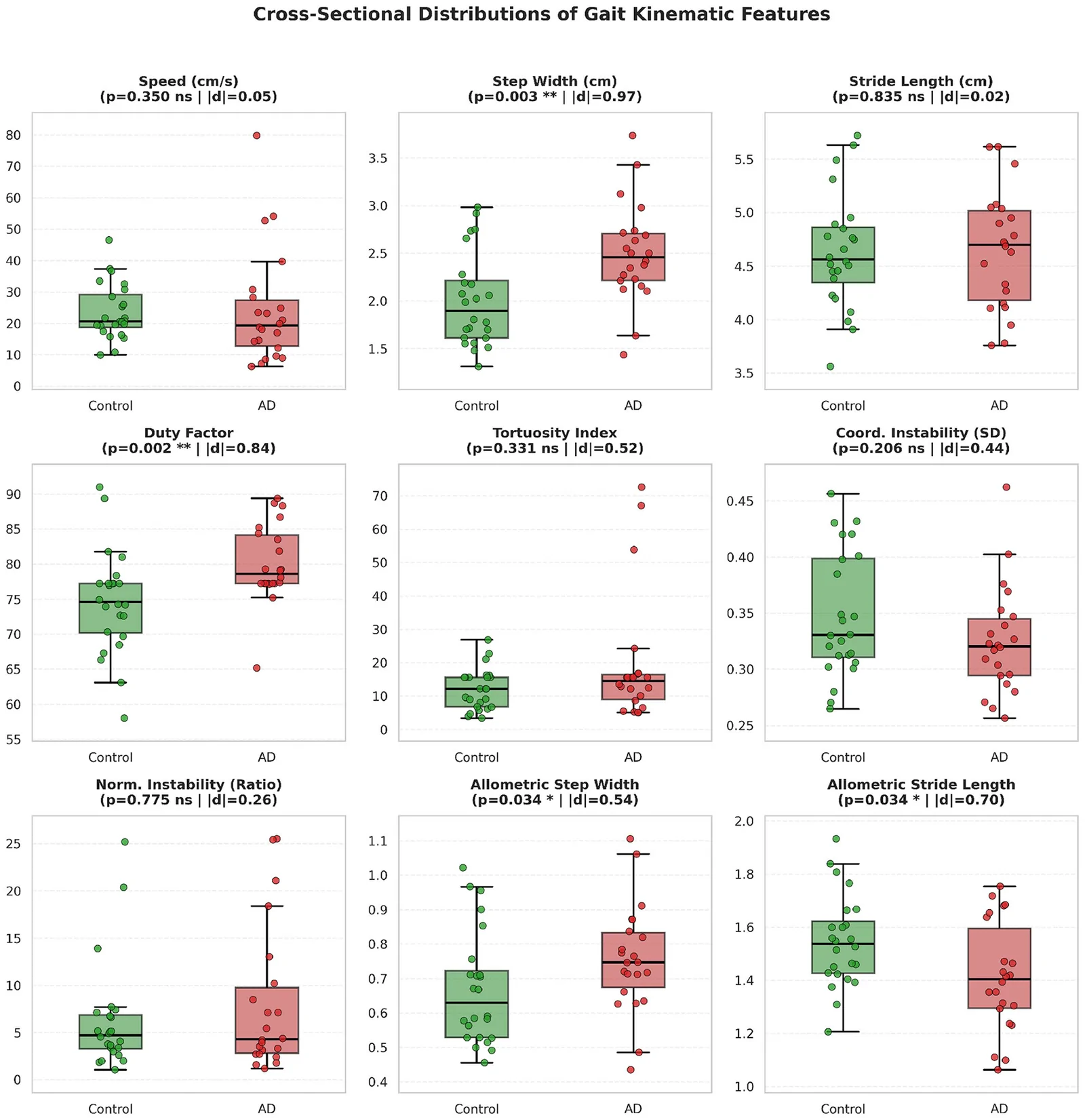
Distribution of cross-sectional gait kinematic features in 3xTg-AD (*n* = 22) and 3xTg-WT control (*n* = 24) mice at 12 months of age. Each panel shows a box plot with individual data points overlaid. Feature titles indicate Mann-Whitney U *p*-values and absolute Cohen's *d* effect sizes. Significant differences are indicated as: ^*^p < 0.05, ^**^p < 0.01.

### 3.2 Univariate analysis of longitudinal gait changes

To contextualize the multivariate predictive modeling, a conventional univariate analysis was performed on the longitudinal change (Δ) for each kinematic feature (Figure 6). The analysis showed that global spatiotemporal metrics, such as Δ Speed and Δ Allometric Step Width, followed comparable trajectories in 3xTg-AD and 3xTg-WT control mice, with non-significant between-group differences (*p*>0.05). In contrast, features related to stride dynamics and coordination showed greater genotype-associated separation. Specifically, the decline in Δ Allometric Stride Length reached statistical significance (*p* = 0.026, |*d*| = 0.85), while coordination-related metrics (Δ Coord. Instability SD and Δ Norm. Instability Ratio) showed increased coordination instability in the 3xTg-AD group and relative stabilization in 3xTg-WT controls. Together, these findings suggest that longitudinal changes in stride length and coordination capture relevant components of genotype-associated locomotor divergence and support their inclusion in the subsequent machine learning framework.

**Figure 6:**
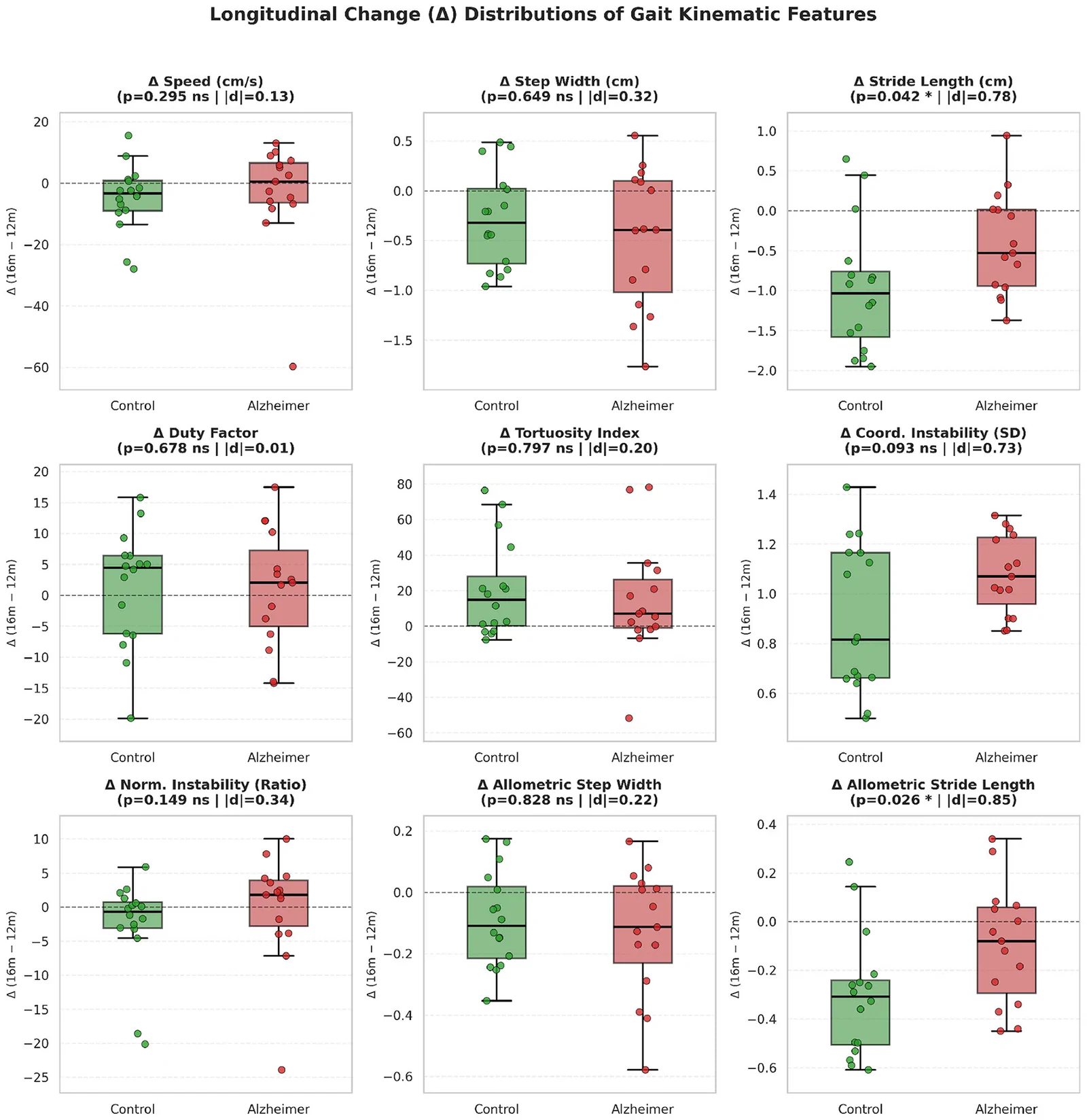
Distribution of longitudinal change (Δ, 16 months minus 12 months) in gait kinematic features for 3xTg-AD (*n* = 15) and 3xTg-WT control (*n* = 16) mice. Each panel displays a box plot with individual data points overlaid. The horizontal dashed line indicates baseline stability or zero change (Δ = 0). Feature titles indicate between-group Mann-Whitney U *p*-values and absolute Cohen's *d* effect sizes. Significant differences are indicated as: ^*^p < 0.05, ^**^p < 0.01.

### 3.3 Experiment 1: Genotype classification at 12 months

The data reported in Table 4 indicate that the Logistic Regression model achieved the highest overall AUC (0.822), with a balanced sensitivity and specificity profile (81.8% and 79.2%, respectively). Although the Extra Trees algorithm obtained a comparable AUC (0.814), it showed a more pronounced imbalance between sensitivity (95.5%) and specificity (58.3%). Therefore, based on its more balanced predictive profile, the Logistic Regression model was selected for the subsequent feature interpretability analysis.

**Table 4:** Performance metrics of the evaluated models for Experiment 1 using the cross-sectional gait feature set.

Model	AUC (bootstrap)	Accuracy (Wilson)	Macro F1 threshold	Sensitivity (Wilson)	Specificity (Wilson)
Logistic Reg.	0.822 ± 0.126	80.4% ± 11.3%	0.479	81.8% ± 15.6%	79.2% ± 15.6%
Extra trees	0.814 ± 0.126	76.1% ± 12.0%	0.424	95.5% ± 10.5%	58.3% ± 18.3%
SVM	0.812 ± 0.133	82.6% ± 10.8%	0.485	77.3% ± 16.7%	87.5% ± 13.3%
LightGBM	0.792 ± 0.139	76.1% ± 12.0%	0.521	77.3% ± 16.7%	75.0% ± 16.5%
CatBoost	0.792 ± 0.133	76.1% ± 12.0%	0.485	81.8% ± 15.6%	70.8% ± 17.1%
MLP neural network	0.792 ± 0.136	78.3% ± 11.7%	0.430	81.8% ± 15.6%	75.0% ± 16.5%
AdaBoost	0.788 ± 0.137	78.3% ± 11.7%	0.497	77.3% ± 16.7%	79.2% ± 15.6%
Naive bayes	0.786 ± 0.126	73.9% ± 12.3%	0.345	68.2% ± 18.2%	79.2% ± 15.6%
Decision tree	0.781 ± 0.138	76.1% ± 12.0%	0.388	63.6% ± 18.7%	87.5% ± 13.3%
Random forest	0.780 ± 0.134	76.1% ± 12.0%	0.497	77.3% ± 16.7%	75.0% ± 16.5%
KNN	0.770 ± 0.124	69.6% ± 12.9%	0.333	59.1% ± 19.0%	79.2% ± 15.6%
XGBoost	0.769 ± 0.140	76.1% ± 12.0%	0.552	68.2% ± 18.2%	83.3% ± 14.6%

Values are reported as point estimates with 95% confidence intervals computed by bootstrap resampling for AUC and Wilson intervals for accuracy, sensitivity, and specificity. Models are ordered by AUC.

#### 3.3.1 ROC curve analysis

Figure 7 illustrates the comparative ROC analysis of the evaluated models using the refined feature set. The Logistic Regression model achieved the highest AUC (0.82), with an operating point, determined by the Macro F1 threshold, positioned relatively close to the ideal coordinate (0,1), indicating a balanced sensitivity-specificity profile. The Support Vector Machine (SVM) and Extra Trees algorithms showed comparable global performance (AUC = 0.81). However, the operating point of the Extra Trees model was shifted toward higher sensitivity with reduced specificity, consistent with the imbalance reported in Table 4. At the lower end of the evaluated models, KNN and XGBoost showed trajectories closer to the chance-level diagonal and yielded the lowest relative AUC values (0.77).

**Figure 7:**
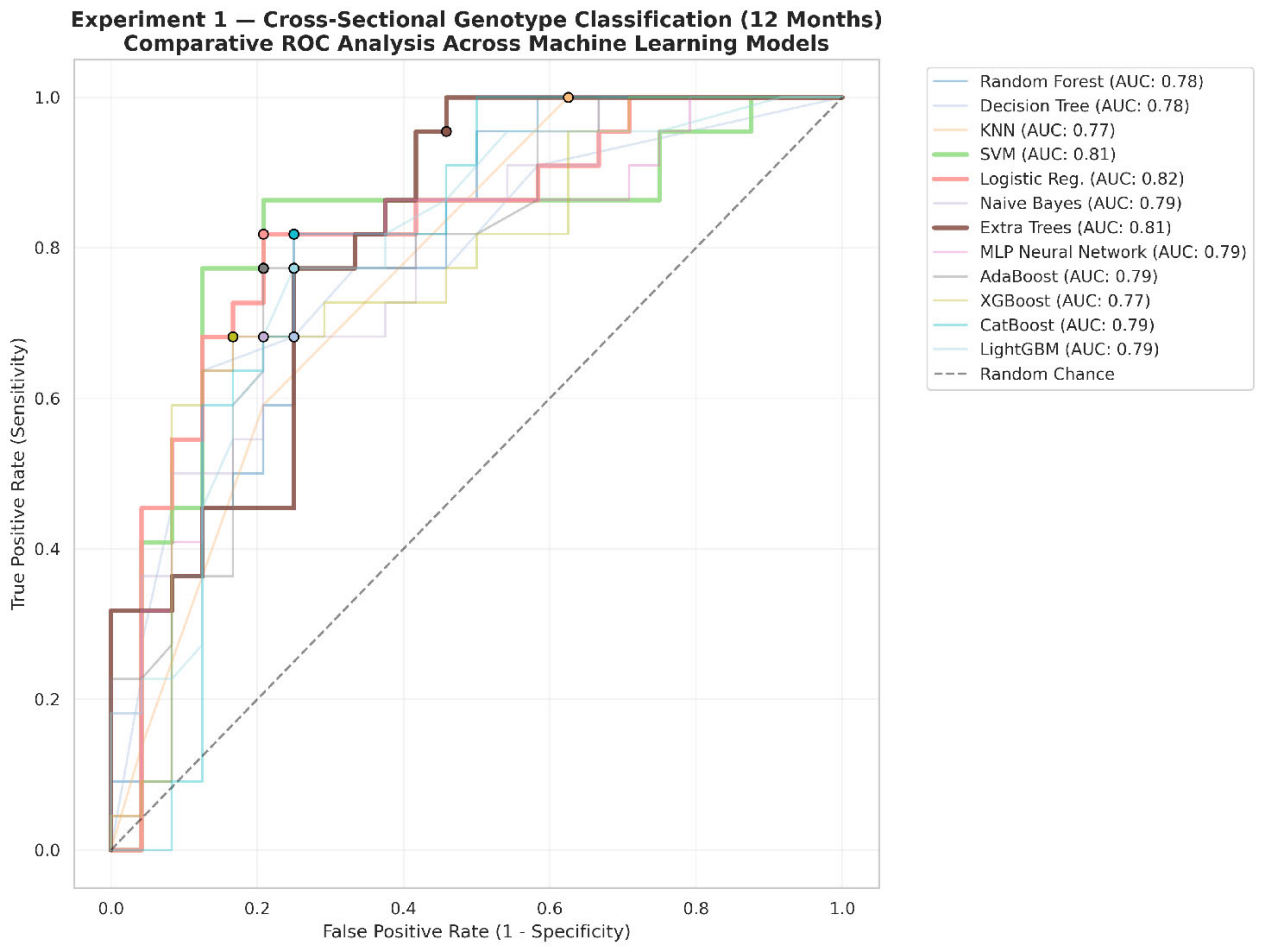
Comparative ROC curves for the evaluated models in Experiment 1 (cross-sectional genotype classification at 12 months of age). Each trajectory represents the ROC profile of a model, with circular markers indicating the operating point at the Macro F1-optimized classification threshold.

#### 3.3.2 Feature importance

The global importance analysis of kinematic features for the first experiment, based on the mean absolute magnitude of SHAP values, is shown in Figure 8. Duty Factor showed the largest mean absolute SHAP contribution within the Logistic Regression model. In descending order of importance, Allometric Stride Length, Allometric Step Width, and Speed emerged as additional relevant contributors to the model output. The remaining variables, including the Tortuosity Index and Normalized Instability Ratio, showed lower contribution magnitudes within the model.

**Figure 8:**
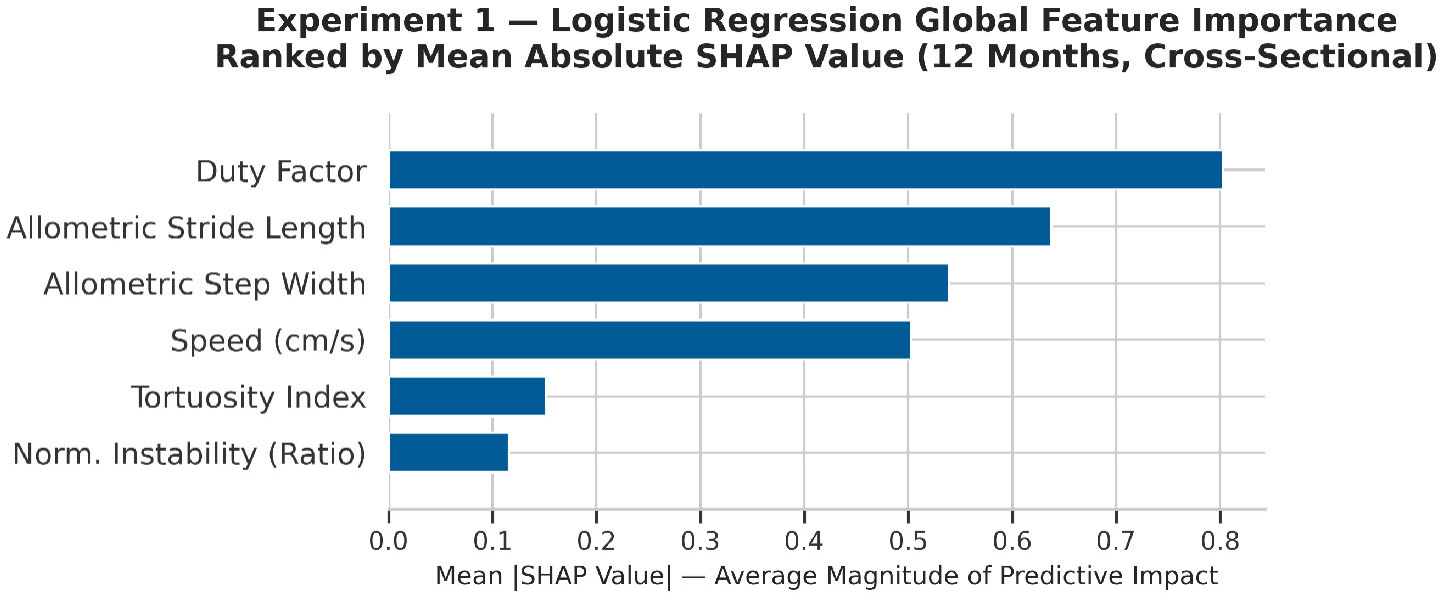
Global feature importance ranking for the logistic regression model in Experiment 1. Features are ordered according to their mean absolute SHAP values, reflecting their relative contribution to the model output.

#### 3.3.3 Model interpretability analysis

Figure 9 illustrates the feature-level contributions of biomechanical variables to the predictions of the Logistic Regression model, evaluated using SHAP. Duty Factor showed the largest contribution to the model output. Higher values of this variable were associated with positive SHAP values (>0), shifting the model output toward the target class (Alzheimer's disease). Allometric Stride Length, ranked as the second most relevant feature, showed an inverse pattern within the model, where lower values were associated with positive SHAP values. Allometric Step Width, ranked third in importance, showed a pattern similar to Duty Factor, with higher values contributing positively to the model output.

**Figure 9:**
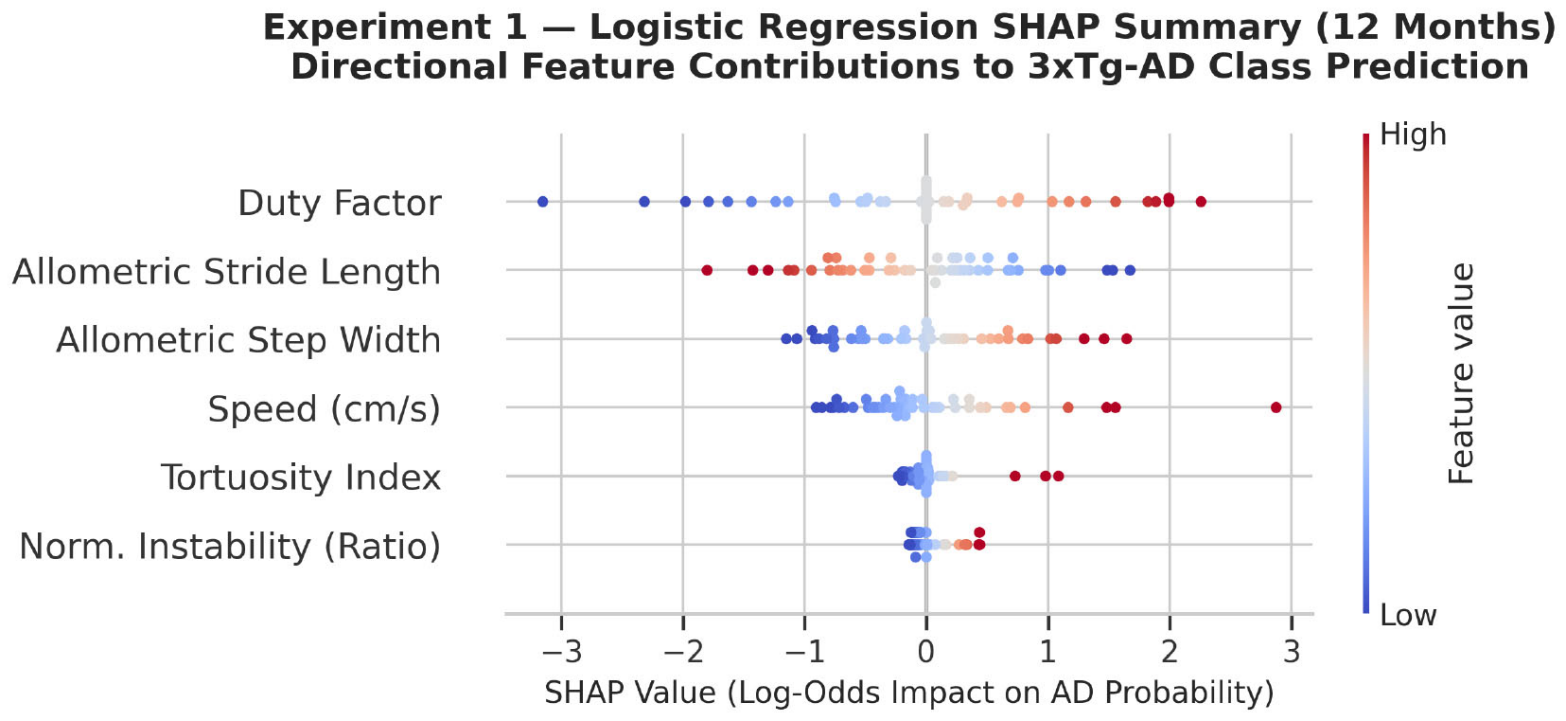
SHAP summary plot for the Logistic Regression model in Experiment 1. Features are ranked by global importance, colors indicate feature values, and horizontal position reflects the contribution of each feature to the model output.

### 3.4 Experiment 2: Longitudinal assessment of gait changes at 16 months

#### 3.4.1 Survival analysis

A Kaplan–Meier survival analysis was performed to evaluate cumulative mortality up to 16 months of age in 3xTg-AD and 3xTg-WT mice, stratified by sex (Figure 10). In males, no significant differences in survival were observed between genotypes (log-rank *p* = 0.236), with comparable survival curves across groups. Similarly, in females, no statistically significant differences were detected (log-rank *p* = 0.759). The number of events was low in both sexes, with a high proportion of censored data at the end of follow-up. Overall, these results indicate that, within the evaluated time window (12–16 months), the 3xTg-AD genotype was not associated with a significant reduction in survival compared with controls. Nevertheless, the attrition of 16 animals between M12 and M16 (Table 1) reduced the paired longitudinal cohort to *N* = 31, which may affect the representativeness of the longitudinal analysis.

**Figure 10:**
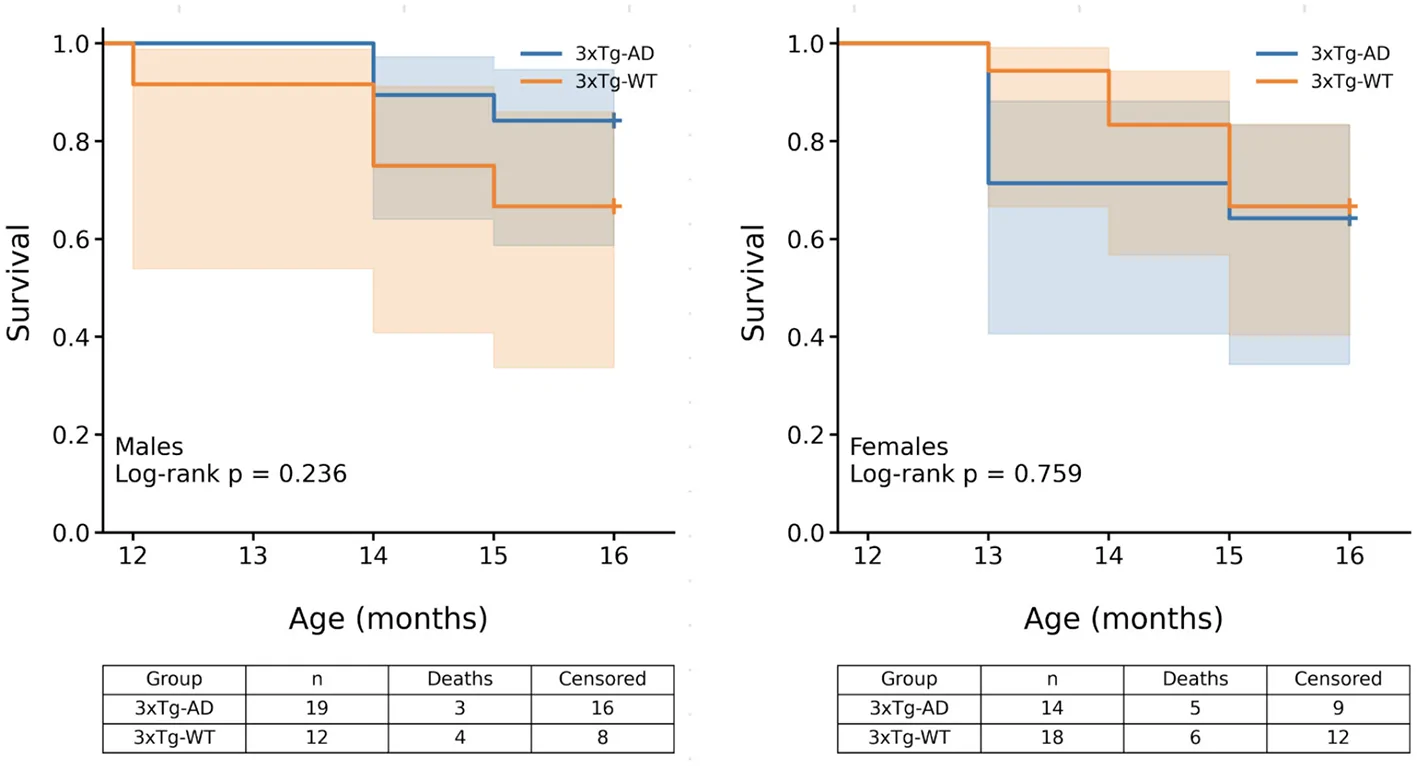
Kaplan–Meier survival curves for 3xTg-AD and 3xTg-WT mice up to 16 months of age, stratified by sex. No significant differences between genotypes were observed in males (*p* = 0.236) or females (*p* = 0.759).

To further evaluate potential attrition bias, baseline 12-month gait features were compared between animals that completed the longitudinal protocol (*n* = 31) and animals lost to follow-up before the 16-month assessment (*n* = 15). A Mann–Whitney U test was applied independently to each spatial and temporal metric. No significant differences were observed across any of the nine kinematic features (all *p*>0.31; rank-biserial |*r*_*rb*_| ≤ 0.187), indicating that baseline locomotor profiles were not detectably different between animals retained in the longitudinal cohort and those lost to follow-up. These results reduce, but do not eliminate, concerns that attrition substantially biased the representativeness of the longitudinal cohort or the interpretation of the locomotor changes observed in Experiment 2.

#### 3.4.2 Longitudinal gait changes following task re-exposure

Longitudinal changes in gait kinematic features were analyzed between the 12- and 16-month assessments in animals that completed the paired follow-up after re-exposure to the locomotor task, with no significant baseline differences found between survivors and animals lost to follow-up (Table 5). Changes were observed across several biomechanical parameters, although their magnitude and direction varied across features and genotypes. Coordination-related variables showed greater temporal variation in 3xTg-AD mice, particularly for Δ Coordination Instability, while reductions in stride-related metrics were also observed. In contrast, global parameters such as Speed showed more comparable changes between genotypes. These findings indicate that longitudinal gait variation in the paired cohort was not uniform across the feature space, with coordination- and stride-related variables showing the most relevant genotype-associated changes after task re-exposure.

**Table 5:** Attrition bias analysis comparing 12-month gait features between animals that completed the longitudinal protocol (*n* = 31) and animals lost to follow-up before the 16-month assessment (*n* = 15).

Feature	Survivor median	Lost-to-follow-up median	*p*-value	*r*_*rb*_
Speed (cm/s)	19.836	21.704	0.349	0.174
Duty factor	77.231	77.231	0.751	-0.060
Tortuosity index	12.257	15.624	0.317	0.185
Norm. instability (Ratio)	4.206	6.610	0.325	0.183
Allometric step width	0.711	0.705	0.851	-0.037
Allometric stride length	1.459	1.547	0.314	0.187
Step width (cm)	2.212	2.154	0.779	-0.054
Stride length (cm)	4.658	4.583	0.542	0.114
Coord. instability (SD)	0.325	0.323	0.907	-0.024

Values are group medians.

No significant baseline differences were detected across the evaluated features.

#### 3.4.3 Model performance evaluation

The predictive performance of the twelve evaluated models is detailed in Table 6, ranked in descending order according to AUC. Overall, classification performance in the longitudinal analysis was modest, with AUC values close to the chance-to-moderate range. The K-Nearest Neighbors (KNN) algorithm achieved the highest AUC (0.627 ± 0.186), but its performance was imbalanced, with higher specificity (75.0%) than sensitivity (46.7%). The Random Forest classifier showed a comparable AUC (0.613 ± 0.206) and a more balanced sensitivity-specificity profile, with an overall accuracy of 61.3%, sensitivity of 60.0%, and specificity of 62.5%. Models such as Logistic Regression, XGBoost, and the MLP Neural Network reached the highest overall accuracy across the cohort (64.5%), but showed lower AUC values (≈0.55–0.58). AdaBoost showed an asymmetric performance profile, with higher sensitivity (80.0%) at the expense of specificity (43.8%). At the lower end of the evaluated models, Naive Bayes and SVM showed AUC values below 0.45. Based on the combination of AUC and balanced sensitivity-specificity performance, Random Forest was selected for subsequent model interpretation.

**Table 6:** Performance metrics of the evaluated models for Experiment 2 using the longitudinal gait-change feature set and a fixed classification threshold of 0.5.

Model	AUC (bootstrap)	Accuracy (Wilson)	Sensitivity (Wilson)	Specificity (Wilson)
KNN	0.627 ± 0.186	61.3% ± 16.2%	46.7% ± 22.5%	75.0% ± 19.7%
Random Forest	0.613 ± 0.206	61.3% ± 16.2%	60.0% ± 22.2%	62.5% ± 21.4%
CatBoost	0.600 ± 0.206	58.1% ± 16.4%	66.7% ± 21.6%	50.0% ± 22.0%
Logistic Reg.	0.583 ± 0.215	64.5% ± 16.0%	60.0% ± 22.2%	68.8% ± 20.7%
LightGBM	0.581 ± 0.203	61.3% ± 16.2%	53.3% ± 22.5%	68.8% ± 20.7%
AdaBoost	0.579 ± 0.212	61.3% ± 16.2%	80.0% ± 19.1%	43.8% ± 21.9%
XGBoost	0.577 ± 0.215	64.5% ± 16.0%	66.7% ± 21.6%	62.5% ± 21.4%
Extra Trees	0.567 ± 0.201	61.3% ± 16.2%	53.3% ± 22.5%	68.8% ± 20.7%
MLP Neural Network	0.550 ± 0.206	64.5% ± 16.0%	66.7% ± 21.6%	62.5% ± 21.4%
Decision Tree	0.548 ± 0.201	54.8% ± 16.5%	60.0% ± 22.2%	50.0% ± 22.0%
Naive Bayes	0.433 ± 0.216	48.4% ± 16.6%	33.3% ± 21.6%	62.5% ± 21.4%
SVM	0.400 ± 0.211	45.2% ± 16.5%	40.0% ± 22.2%	50.0% ± 22.0%

Values are reported as point estimates with 95% confidence intervals computed by bootstrap resampling for AUC and Wilson intervals for accuracy, sensitivity, and specificity. Models are ordered by AUC.

#### 3.4.4 ROC curve analysis

Figure 11 presents the ROC curves of the evaluated models in the longitudinal predictive framework. Overall, the curves showed a step-like pattern, likely reflecting the restricted sample size of the paired longitudinal cohort. The K-Nearest Neighbors (KNN) algorithm showed the highest AUC (0.627), but its operating point at the fixed 0.5 classification threshold was associated with lower sensitivity and higher specificity. In contrast, the Random Forest model (AUC = 0.613) showed a more balanced operating point, consistent with the sensitivity-specificity profile reported in Table 6. AdaBoost showed an asymmetric operating profile, with higher sensitivity but a higher false-positive rate. Together, these results indicate that the longitudinal classification task showed limited discriminative performance, with Random Forest providing the most balanced operating profile among the evaluated models.

**Figure 11:**
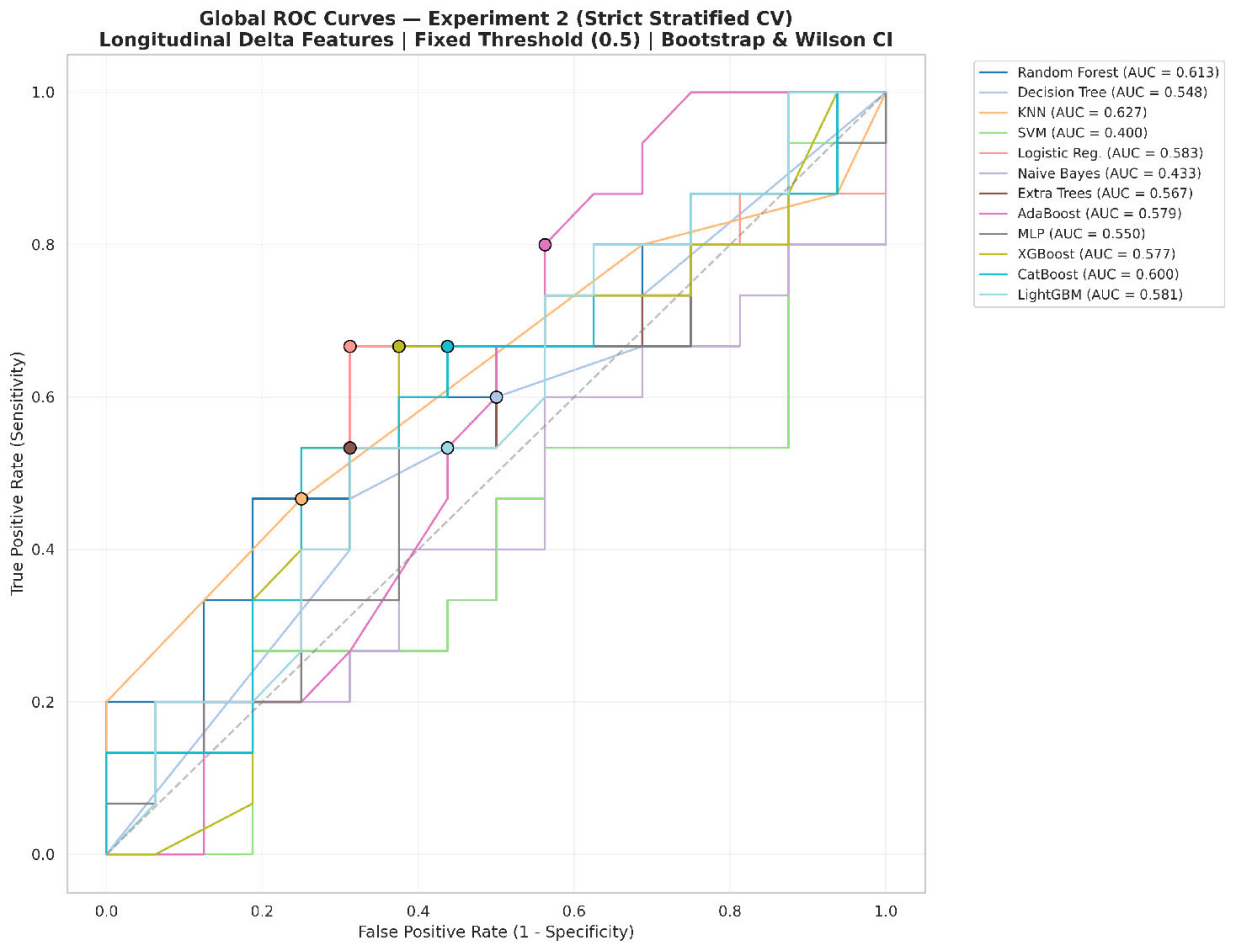
Comparative ROC curves for the evaluated models in Experiment 2 (longitudinal gait-change classification). Each trajectory represents the ROC profile of a model, with circular markers indicating the operating point at a fixed classification threshold of 0.5.

#### 3.4.5 Feature importance analysis

SHAP-based interpretability analysis of the Random Forest model indicated that the longitudinal model output was mainly associated with two kinematic features (Figure 12). The largest mean absolute SHAP contribution corresponded to the longitudinal variation in stride length (Δ Allometric Stride Length). The second largest contribution corresponded to the longitudinal variation in dynamic coordination (Δ Norm. Instability Ratio). The remaining variables, including Δ Speed, Δ Duty Factor, Δ Allometric Step Width, and Δ Tortuosity Index, showed lower contribution magnitudes within the model.

**Figure 12:**
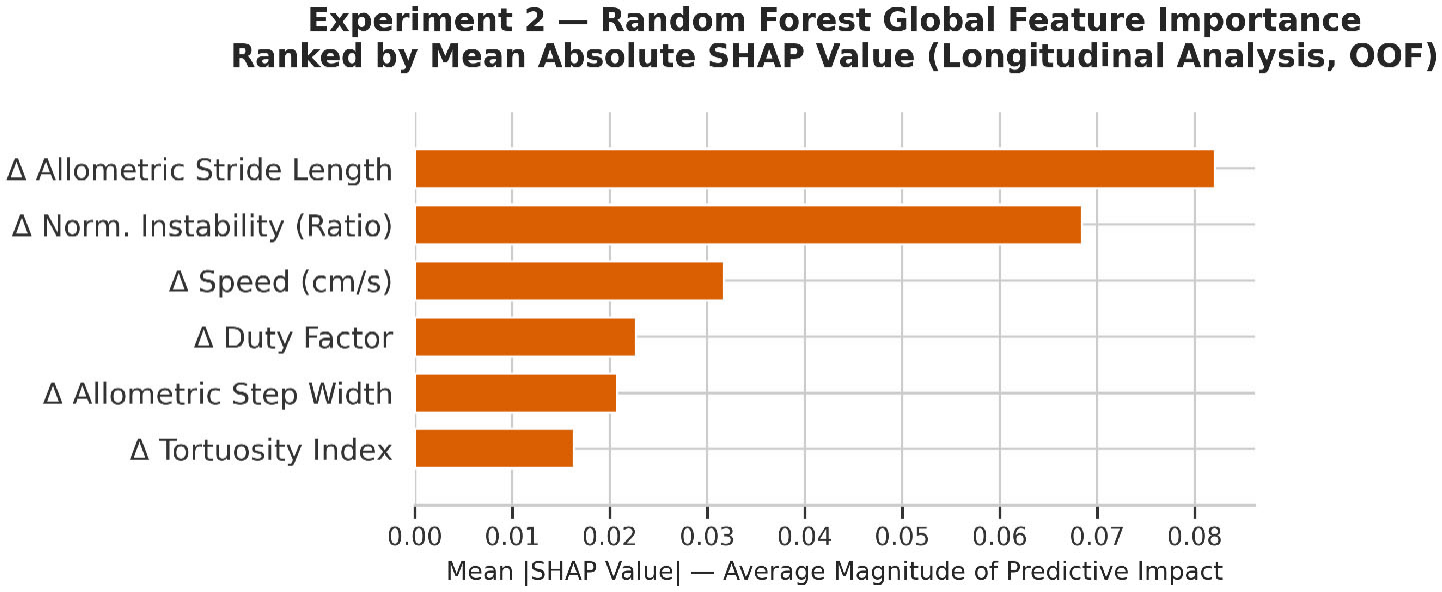
SHAP-based global feature contribution ranking for the longitudinal Random Forest model in Experiment 2. Features are ordered according to their mean absolute SHAP values, computed from out-of-fold predictions.

#### 3.4.6 Longitudinal SHAP analysis

The SHAP beeswarm analysis (Figure 13) illustrates the directional contribution of longitudinal feature changes (Δ) to the output of the Random Forest model. The distribution indicates that Δ Allometric Stride Length, the highest-ranked feature, showed a positive association within the model: higher Δ values were associated with positive SHAP values, shifting the model output toward the 3xTg-AD class. Similarly, higher values of Δ Norm. Instability Ratio were associated with positive SHAP values, suggesting that longitudinal increases in coordination instability contributed to genotype classification within the model. In contrast, lower-ranked global metrics, such as Δ Speed, showed more centered or dispersed SHAP distributions along the horizontal axis, indicating a weaker and less consistent contribution to the model output.

**Figure 13:**
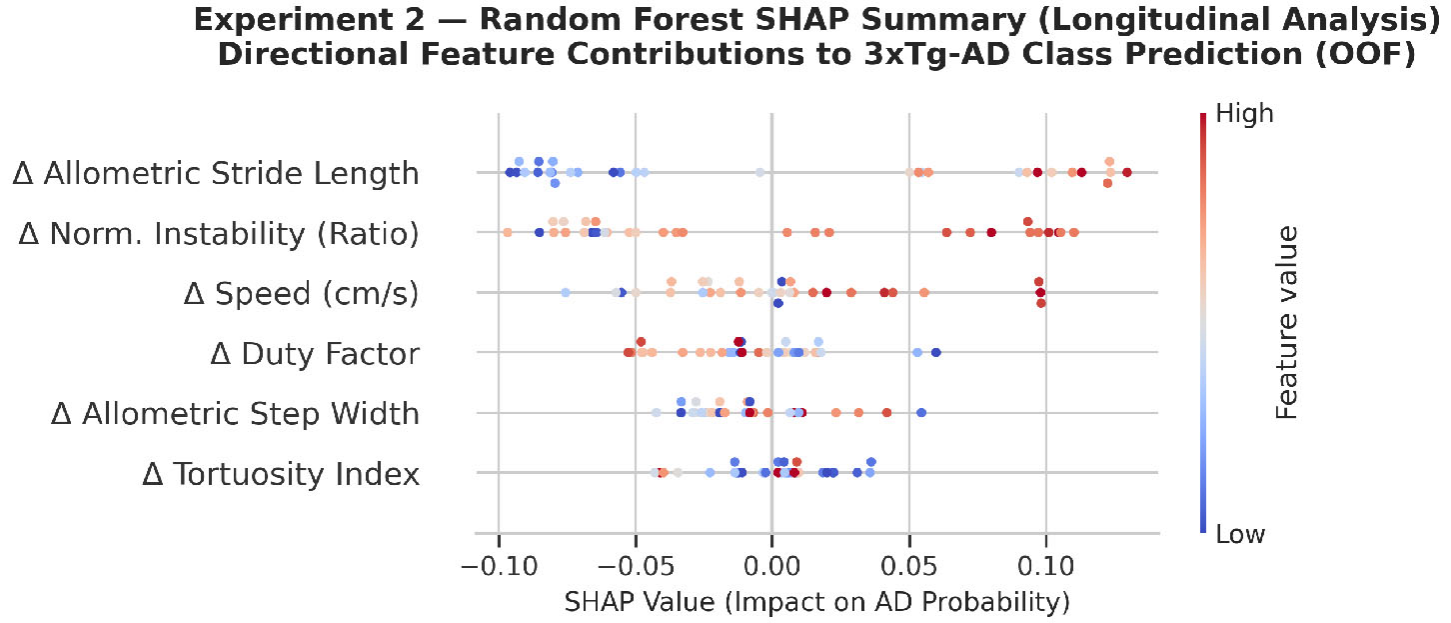
SHAP beeswarm summary plot for the longitudinal Random Forest model in Experiment 2. The plot illustrates the direction and magnitude of contributions from kinematic variations (Δ) to the model output, with colors indicating normalized feature values (blue for low or decreased values, red for high or increased values).

## 4 Discussion

In the cross-sectional analysis performed at 12 months of age, discrimination between 3xTg-AD mice and 3xTg-WT controls was associated with a distributed locomotor phenotype involving temporal, spatial, and motor-control-related variables. In contrast, the longitudinal analysis between 12 and 16 months showed more limited classification performance, although changes in Allometric Stride Length and Normalized Coordination Instability contributed meaningfully to the locomotor divergence observed in 3xTg-AD mice. Overall, these results indicate that computational gait analysis may provide useful information for characterizing locomotor alterations in preclinical models of Alzheimer's disease, provided that the findings are interpreted cautiously in light of the sample size, the absence of external validation, and the inherent limitations of the longitudinal design.

In the cross-sectional analysis, Logistic Regression showed the most balanced predictive performance among the evaluated models. Although some ensemble methods reached comparable AUC values, they exhibited more pronounced imbalances between sensitivity and specificity, suggesting greater susceptibility to biased classification strategies in the context of a small cohort. The relative stability of Logistic Regression suggests that, at this intermediate stage of the model, genotype-related locomotor differences can be represented by a linear combination of kinematic variables. From a biomechanical perspective, this pattern indicates that early gait alterations are not explained by a single dimension, but rather by the joint contribution of variables such as Duty Factor, Allometric Stride Length, Allometric Step Width, and Speed.

The conventional statistical analyses complemented this interpretation by showing group-level differences mainly in variables related to the base of support, the temporal distribution of stance phase, and body-mass-adjusted spatial parameters. These differences are consistent with the notion that gait in the 3xTg-AD model reflects a complex functional phenotype, in which spatiotemporal, postural, and motor-control alterations may coexist before the emergence of severe locomotor deficits. In this sense, the combination of univariate analysis and machine learning provided two complementary levels of interpretation: first, by identifying variables that differed statistically between groups, and second, by evaluating how these variables contributed jointly within a multivariate classification model.

The longitudinal analysis presented a more complex scenario. Unlike the cross-sectional analysis, models applied to changes between 12 and 16 months achieved more moderate predictive performance. This outcome likely reflects the simultaneous influence of multiple factors, including physiological aging, genotype-associated effects, selective survival, and re-exposure to the locomotor task. In particular, some global gait changes, such as Speed and certain spatial parameters, followed similar trajectories in 3xTg-AD and 3xTg-WT mice, suggesting that part of the longitudinal variation may be related to normal aging or to general effects of repeated task exposure.

Despite the moderate classification performance in the longitudinal analysis, the results suggest that variables related to stride length and interlimb coordination may capture relevant components of locomotor change associated with the 3xTg-AD genotype. In particular, longitudinal variation in Allometric Stride Length and Normalized Coordination Instability emerged as important components in both the statistical analysis and model interpretation. This pattern suggests that, while global metrics may be influenced by common aging-related processes, coordination-related and dynamic-organization variables may be more sensitive to functional alterations in the 3xTg-AD model.

From a neurobiological perspective, the observed coordination-related alterations are compatible with the notion that gait depends on distributed networks integrating motor control, postural stability, spatial planning, and cognitive components. In Alzheimer's disease models, disruption of these networks may manifest not only as changes in speed or stride length, but also as increased variability in interlimb coordination. In this context, Normalized Coordination Instability may be interpreted as a functional descriptor of the temporal stability of the locomotor pattern. Nevertheless, the present study does not incorporate direct neuropathological or neurophysiological measurements; therefore, a causal relationship between the identified kinematic variables and specific mechanisms of neurodegeneration cannot be established.

SHAP-based interpretation allowed us to identify which variables contributed most strongly to model output. In the cross-sectional analysis, the main contributions were distributed across temporal and spatial variables, whereas in the longitudinal analysis they were concentrated on changes in stride length and coordination. While SHAP successfully isolated the most discriminative variables, these values explain the internal decision structure of the model. Therefore, the highlighted features should be understood as highly relevant computational markers for classification within this dataset, rather than confirmed pathogenic mechanisms. This distinction is particularly important in studies with small samples, where the stability of feature-contribution hierarchies may depend on cohort composition.

The use of markerless pose estimation represents a methodological strength of this study, as it enables non-invasive quantification of locomotor patterns with minimal physical restriction of the animals. The incorporation of DeepLabCut allowed the extraction of two-dimensional coordinates from relevant anatomical keypoints and the derivation of kinematic variables related to speed, support, stride, tortuosity, and coordination. In addition, the inclusion of operational definitions for the extracted variables, the sensitivity analysis of the confidence threshold, and the characterization of tracking quality strengthen the reproducibility of the pipeline. In particular, the justification of the selected confidence threshold provides a balance between noise reduction and the preservation of potentially relevant locomotor events, especially during rapid limb swing phases.

Several limitations should be acknowledged. First, the size of the pose-estimation training set was relatively limited, with 300 manually annotated frames. Although the overall model performance was compatible with sub-centimeter precision under the applied calibration, a larger and biologically balanced training set could improve tracking confidence, particularly during rapid or partially occluded events. Future studies should increase the number of annotated frames per condition, incorporate greater postural diversity, and perform additional manual validation of intermittent locomotor events.

Second, the analysis relied exclusively on two-dimensional data obtained from a ventral-view recording configuration. This setup is useful for capturing contact points, base of support, and spatiotemporal displacements, but it limits the evaluation of three-dimensional gait components, such as vertical limb clearance, joint angles, foot clearance, and out-of-plane postural oscillations. Therefore, the results should be interpreted as a 2D characterization of locomotor organization. Future studies should incorporate multi-camera systems or 3D reconstruction to provide a more complete assessment of gait biomechanics in models of neurodegeneration.

Third, the sample size and unequal sex distribution limited the ability to evaluate sex-specific effects. Although sex-stratified analysis would be relevant given the evidence of sex-related differences in the progression of the 3xTg-AD model, the current cohort composition did not allow stable classifiers to be trained within each subgroup. For this reason, supervised models were applied to the pooled cohort. This decision increased the available sample size for the global analysis but reduced the ability to determine whether the identified locomotor features are equivalent in males and females. Future studies should prioritize cohorts balanced by sex and genotype to evaluate the stability of these patterns.

Fourth, the longitudinal design did not include an independent non-re-exposed control group. Consequently, the changes observed between 12 and 16 months cannot be attributed exclusively to disease progression. These changes may reflect an interaction among neuropathological progression, physiological aging, selective survival, habituation, residual motor learning, and re-exposure to the recording system. Although the attrition-bias analysis did not reveal significant baseline differences between surviving and non-surviving animals, the reduction of the longitudinal cohort remains a potential source of uncertainty. Therefore, the results of Experiment 2 should be considered exploratory and require confirmation through longitudinal designs including parallel exposed and non-re-exposed groups.

Additionally, the results should be interpreted considering that the study was conducted in a single experimental cohort. Although cross-validation allowed internal model performance to be estimated under a controlled framework, the stability of predictive performance and feature-contribution hierarchies should be evaluated in future independent cohorts. Replication in additional experimental groups, ideally using comparable acquisition settings and larger cohorts, will be necessary to determine the generalizability of the proposed approach more precisely.

Finally, this study focuses on the computational characterization of gait and does not include direct neuropathological or neurophysiological measurements. Therefore, the observed associations between kinematic variables and genotype do not allow causal mechanisms to be established. Future integration of gait analysis with markers of amyloid burden, tau pathology, neuroinflammation, synaptic alterations, or neurophysiological recordings would help determine more precisely whether changes in locomotor coordination reflect specific processes of neural dysfunction.

In conclusion, computational gait analysis provides relevant, multidimensional insights into locomotor alterations in the 3xTg-AD mouse model. At 12 months of age, genotype-related differences manifest as a distributed pattern of spatiotemporal and motor-control variations. Longitudinally, while classification performance is more modest, reductions in Allometric Stride Length and increases in Normalized Coordination Instability emerge as primary indicators of locomotor divergence. These findings demonstrate that integrating markerless pose estimation with machine learning can successfully operationalize gait as a computational motor biomarker, providing a robust complementary tool for characterizing complex behavioral phenotypes in preclinical models of Alzheimer's disease.

## Data Availability

The datasets generated and analyzed during the current study, including extracted kinematic features and analysis scripts, are available from the corresponding author upon reasonable request. The machine learning pipeline code is available at: https://github.com/ElXylo/3xTgAD-gait-kinematics.
