# Supplementary material for: Computational gait analysis in the 3xTg-AD mouse model: evidence of progressive alterations in locomotor coordination
Source: Front Aging Neurosci. 2026 Sep 23;18:1879487. doi: 10.3389/fnagi.2026.1879487 (PMC13642803; doi:10.3389/fnagi.2026.1879487)
Supplement: Supplementary file 1 [file Data_Sheet_1.pdf]

Supplementary Material

**Model**	**Evaluated Hyperparameter Grid**
Random Forest	`n_estimators`: [100, 300], `criterion`: ['gini', 'entropy'], `max_depth`: [3, 5, 7], `min_samples_split`: [2, 5], `min_samples_leaf`: [2, 4], `max_features`: ['sqrt', 'log2']
Decision Tree	`criterion`: ['gini', 'entropy'], `max_depth`: [2, 3], `min_samples_split`: [4, 6, 10], `min_samples_leaf`: [3, 4], `max_features`: ['sqrt', 'log2']
KNN	`n_neighbors`: [3, 5, 7, 9, 11], `weights`: ['uniform', 'distance'], `metric`: ['euclidean', 'manhattan']
SVM	`C`: [0.1, 1, 10, 100], `kernel`: ['linear', 'rbf'], `gamma`: ['scale', 0.1, 0.01, 0.001]
Logistic Regression	`C`: [0.01, 0.1, 1, 10, 100], `penalty`: ['l1', 'l2'], `solver`: ['liblinear']
Naive Bayes	`var_smoothing`: 100 logarithmically spaced values $\in [10^{-9}, 10^{0}]$
XGBoost	`n_estimators`: [50, 100, 200], `learning_rate`: [0.01, 0.1, 0.2], `max_depth`: [3, 4, 5], `subsample`: [0.8, 1.0], `colsample_bytree`: [0.8, 1.0]
CatBoost	`iterations`: [100, 200], `depth`: [4, 6], `learning_rate`: [0.01, 0.05], `l2_leaf_reg`: [1, 3, 5]
LightGBM	`n_estimators`: [50, 100], `learning_rate`: [0.01, 0.05, 0.1], `num_leaves`: [5, 7, 10], `max_depth`: [2, 3, 4], `min_child_samples`: [2, 5]
AdaBoost	`n_estimators`: [50, 100, 200, 300], `learning_rate`: [0.01, 0.05, 0.1, 0.5, 1.0], `estimator__max_depth`: [1, 2, 3], `estimator__criterion`: ['gini', 'entropy'], `estimator__min_samples_leaf`: [2, 4]
Extra Trees	`n_estimators`: [100, 200, 300], `criterion`: ['gini', 'entropy'], `max_depth`: [3, 5, 7, None], `min_samples_leaf`: [2, 4], `max_features`: ['sqrt', 'log2'], `bootstrap`: [False, True], `class_weight`: ['balanced', 'balanced_subsample']
MLP Neural Network	`hidden_layer_sizes`: [(8,), (16,), (32,)], `activation`: ['tanh', 'relu'], `alpha`: [1.0, 10.0, 100.0]

Table Supplementary Table 1: Hyperparameter search space evaluated via nested grid search for all supervised machine learning models in Experiment 1. All models used `scoring=f1_macro` and 5-fold `StratifiedKFold` cross-validation.

Optimal operating thresholds were determined contextually for each experimental design. For Experiment 1, the classification threshold was calibrated by maximizing the Macro F1 score within a restricted analytical range ($[0.2, 0.8]$), incorporating a stability constraint that reverted the threshold to the standard 0.5 if the predicted probability distribution collapsed (standard deviation < 0.05). Conversely, for Experiment 2, to prevent any form of post-hoc

Model	Evaluated Hyperparameter Grid
Random Forest	`n_estimators`: [100, 200], `max_depth`: [3, 5], `min_samples_leaf`: [2, 4], `max_features`: ['sqrt']
Decision Tree	`criterion`: ['gini', 'entropy'], `max_depth`: [2, 3], `min_samples_split`: [4, 6], `min_samples_leaf`: [3, 4]
KNN	`n_neighbors`: [3, 5, 7], `weights`: ['uniform', 'distance'], `metric`: ['euclidean', 'manhattan']
SVM	`C`: [0.1, 1, 10, 50], `kernel`: ['linear', 'rbf'], `gamma`: ['scale', 0.1, 0.01]
Logistic Regression	`C`: [0.01, 0.1, 1, 10, 100], `penalty`: ['l1', 'l2'], `solver`: ['liblinear']
Naive Bayes	`var_smoothing`: 100 logarithmically spaced values $\in [10^{-9}, 10^{0}]$
XGBoost	`n_estimators`: [50, 100], `learning_rate`: [0.01, 0.1], `max_depth`: [2, 3, 4], `subsample`: [0.8, 1.0]
CatBoost	`iterations`: [50, 100], `depth`: [3, 4], `learning_rate`: [0.01, 0.05], `l2_leaf_reg`: [1, 3, 5]
LightGBM	`n_estimators`: [50, 100], `learning_rate`: [0.01, 0.05], `num_leaves`: [3, 7], `max_depth`: [2, 3], `min_child_samples`: [2, 5]
AdaBoost	`n_estimators`: [50, 100, 200, 300], `learning_rate`: [0.01, 0.05, 0.1, 0.5, 1.0]
Extra Trees	`n_estimators`: [50, 100, 200], `criterion`: ['gini', 'entropy'], `max_depth`: [2, 3, 5, None], `min_samples_split`: [2, 4], `min_samples_leaf`: [1, 2], `class_weight`: ['balanced', 'balanced_subsample']
MLP Neural Network	`hidden_layer_sizes`: [(8,), (16,), (32,)], `activation`: ['tanh', 'relu'], `alpha`: [1.0, 10.0, 100.0]

Table Supplementary Table 2: Hyperparameter search space evaluated via nested grid search for the longitudinal classification models in Experiment 2. All models used `scoring=f1_macro` and 5-fold `StratifiedKFold` cross-validation.

Model	Optimal Hyperparameters
Logistic Regression	C: 1; penalty: l2; solver: liblinear
Random Forest	criterion: gini; max_depth: 3; max_features: sqrt; min_samples_leaf: 2; min_samples_split: 2; n_estimators: 300
Extra Trees	bootstrap: False; class_weight: balanced; criterion: gini; max_depth: 3; max_features: sqrt; min_samples_leaf: 2; n_estimators: 300
SVM	C: 1; gamma: scale; kernel: linear
LightGBM	learning_rate: 0.01; max_depth: 3; min_child_samples: 5; n_estimators: 50; num_leaves: 5
CatBoost	depth: 6; iterations: 100; l2_leaf_reg: 1; learning_rate: 0.01
MLP Neural Network	activation: tanh; alpha: 1.0; hidden_layer_sizes: (8,)
AdaBoost	estimator__criterion: gini; estimator__max_depth: 1; estimator__min_samples_leaf: 2; learning_rate: 0.05; n_estimators: 300
Naive Bayes	var_smoothing: 0.351
Decision Tree	criterion: gini; max_depth: 3; max_features: sqrt; min_samples_leaf: 4; min_samples_split: 4
KNN	metric: manhattan; n_neighbors: 3; weights: uniform
XGBoost	colsample_bytree: 0.8; learning_rate: 0.01; max_depth: 4; n_estimators: 100; subsample: 0.8

Table Supplementary Table 3: Selected hyperparameter configurations for the classification models in Experiment 1. Hyperparameters were selected using grid search within the cross-validation pipeline.

Model	Optimal Hyperparameters
Random Forest	max_depth: 3; max_features: sqrt; min_samples_leaf: 4; n_estimators: 200
Decision Tree	criterion: gini; max_depth: 3; min_samples_leaf: 4; min_samples_split: 4
KNN	metric: manhattan; n_neighbors: 7; weights: uniform
SVM	C: 1; gamma: scale; kernel: linear
Logistic Regression	C: 1; penalty: l2; solver: liblinear
Naive Bayes	var_smoothing: 0.285
XGBoost	learning_rate: 0.01; max_depth: 3; n_estimators: 50; subsample: 1.0
CatBoost	depth: 4; iterations: 50; l2_leaf_reg: 5; learning_rate: 0.01
LightGBM	learning_rate: 0.01; max_depth: 3; min_child_samples: 2; n_estimators: 50; num_leaves: 7
AdaBoost	learning_rate: 0.01; n_estimators: 200
Extra Trees	class_weight: balanced; criterion: gini; max_depth: None; min_samples_leaf: 1; min_samples_split: 2; n_estimators: 200
MLP Neural Network	activation: tanh; alpha: 1.0; hidden_layer_sizes: (16,)

Table Supplementary Table 4: Selected hyperparameter configurations for the longitudinal classification models in Experiment 2. Hyperparameters were selected using grid search within the cross-validation pipeline.

threshold optimization bias on the restricted longitudinal sample ($N = 31$), a fixed classification threshold of 0.5 was enforced uniformly across all algorithms. Final predictive performance was quantified using AUC, accuracy, sensitivity, and specificity. All metrics are reported with their corresponding 95% confidence intervals, computed via bootstrap resampling for the AUC and the Wilson score interval for categorical proportions [**?**].
